# Supplementary material for: Structures of the EphA2 Receptor at the Membrane: Role of Lipid Interactions
Source: Structure. 2016 Feb 2;24(2):337–47. doi: 10.1016/j.str.2015.11.008 (PMC4744086; doi:10.1016/j.str.2015.11.008)
Supplement: Document S1. Figures S1–S6, Table S1, and Supplemental Information [file mmc1.pdf]

Structure, Volume *24*

Supplemental Information

Structures of the EphA2 Receptor at the Membrane: Role of Lipid Interactions

Matthieu Chavent, Elena Seiradake, E. Yvonne Jones, and Mark S.P. Sansom

Supplemental Information

Supplementary text related to Method and Figures 2, 6, S3, S4 and S6

In order to quantify the protein-lipids interactions occurring during the simulations, we first define an interaction count function, $C_l(res_i)$of the interactions between residue i and the lipid type l for a time *t*:

$$C_l(res_i) = \begin{cases} \text{Nb. atoms from lipid type } l \text{ within } 8\text{ Å of } res_i \\ 0 \text{ otherwise} \end{cases}$$

The proportional interaction of a residue i for a lipid type l , $I_{i,l}$, is then obtained summation of the interaction counts over the time the protein forms a stable interaction with the membrane and normalising it by the duration of the interaction:

$$I_{i,l} = \frac{1}{N - n_b} \sum_{n_b}^{N} C_l(res_i)$$

where *N* is the total simulation time and n_b the time at which the interaction occurs.

This allows one to define a proportional interaction for PC and PG lipids (respectively $I_{i,POPC}$ and $I_{i,POPG}$) for a residue i.

We then normalized over the different residues using the maximum of PC+PG value to obtain a value between 0 and 1:

$$Max_{I(PC,PG)} = \max(I_{i,POPC} + I_{i,POPG})$$

with *i* varying from first residue number to the last.

$$N(I_i) = \frac{I_{i,POPC}}{Max_{I(PC,PG)}} + \frac{I_{i,POPG}}{Max_{I(PC,PG)}}$$

We used $N(I_i)$ values to create the histograms presented in Figures 2, 6, S3, S4 and S6.

The curve, highlighting the preference for PG lipid was obtained using the expression:

$$P_{i,POPG} = \frac{I_{i,POPG}}{Max_{I(PC,PG)}} - \frac{I_{i,POPC}}{Max_{I(PC,PG)}}$$

Table S1 related to Table 1: Summary of the FN2 simulations interacting with the membrane. Simulation duration and time to binding for the diffusion protocol (Wild Type – WT – and mutant). The 'time to binding' refers to the time elapsed between the start of the simulation and the onset of a stable interaction with the membrane. Each simulation was run for at least 500 ns of simulation following formation of this initial interaction. If the protein did not interact with the the membrane after 6 μs it was considered not to bind.

System name	duration (ns)	binding	time to binding (ns)
**100% PC (WT)**			
FN2 180°	6000	No	###
FN2 0°	6000	No	###
FN2 90° A	6000	No	###
FN2 90° B	6000	yes	1430
**60% PC - 40% PG (FN2 WT)**			
FN2 180°	2000	yes	1525
FN2 0°	2000	yes	1500
FN2 90° A	1000	yes	147
FN2 90° B	1000	yes	107
**60% PC - 40% PG (FN2 K441E+K443E mutant)**			
FN2 180°	1000	Yes	324
FN2 0°	3000	Yes	1934
FN2 90° A	2000	Yes	1400
FN2 90° B	3000	Yes	2150
**60% PC - 40% PG (FN2 K441E+K443E+R465E mutant)**			
FN2 180°	6000	No	###
FN2 0°	1000	Yes	343
FN2 90° A	5000	Yes	4198
FN2 90° B	2000	Yes	990
**60% PC - 40% PG (FN2 K441E+K443E+R465E+F490A mutant)**			
FN2 180°	2000	Yes	1672
FN2 0°	4000	Yes	3121
FN2 90° A	6000	No	###
FN2 90° B	2000	No	###
**60% PC - 40% PG (FN2 K441E+K443E+R465E+F490A+W467A mutant)**			
FN2 180°	2000	Yes	1498
FN2 0°	7000	Yes	5759
FN2 90° A	7000	Yes	5995
FN2 90° B	2000	Yes	1095

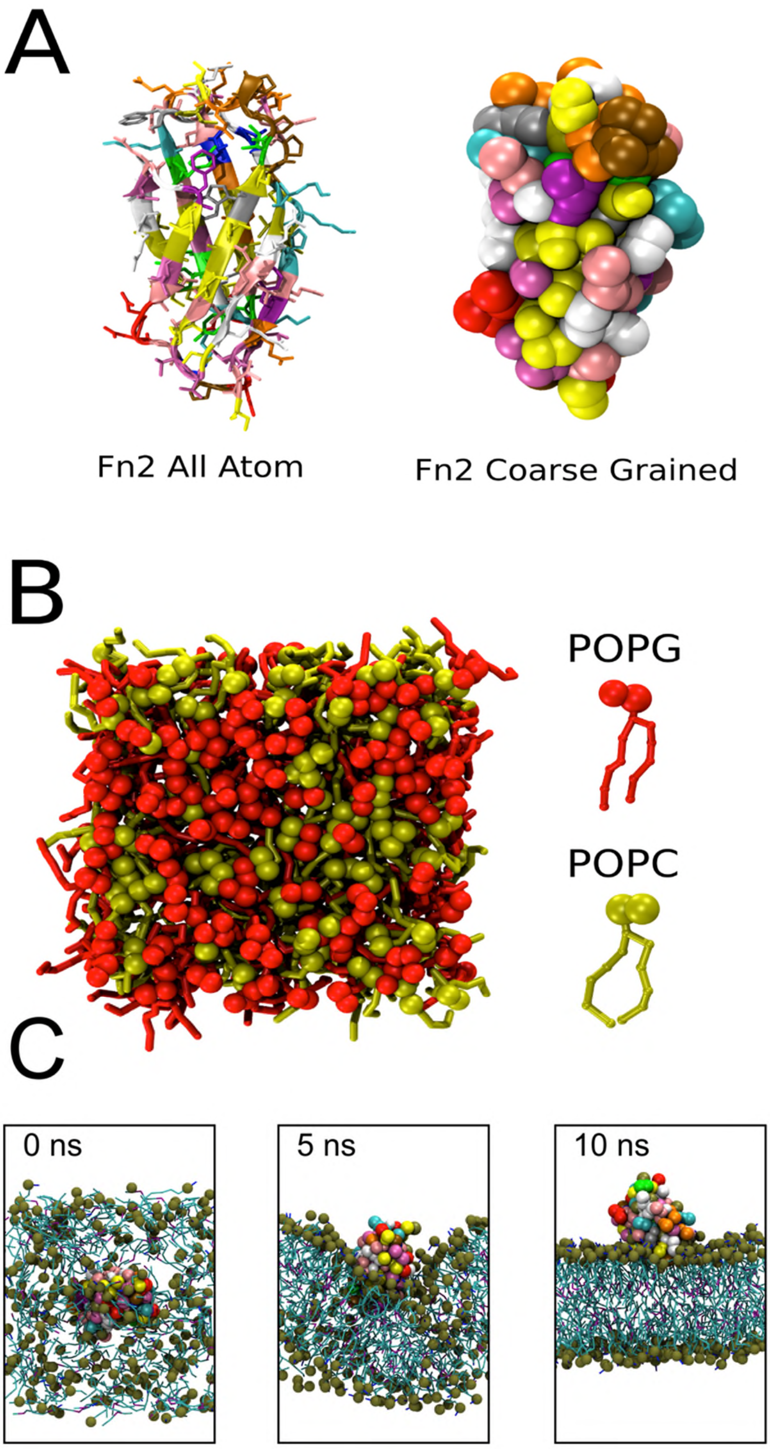

Figure S1 (related to Figure 1 and Methods): (A) Atomistic (left) and coarse-grained (right) models of the FN2 domain. (B) Mixed (PG+PC) membrane model for CG simulations. (C) Progress of the bilayer self-assembly simulation at different time steps.

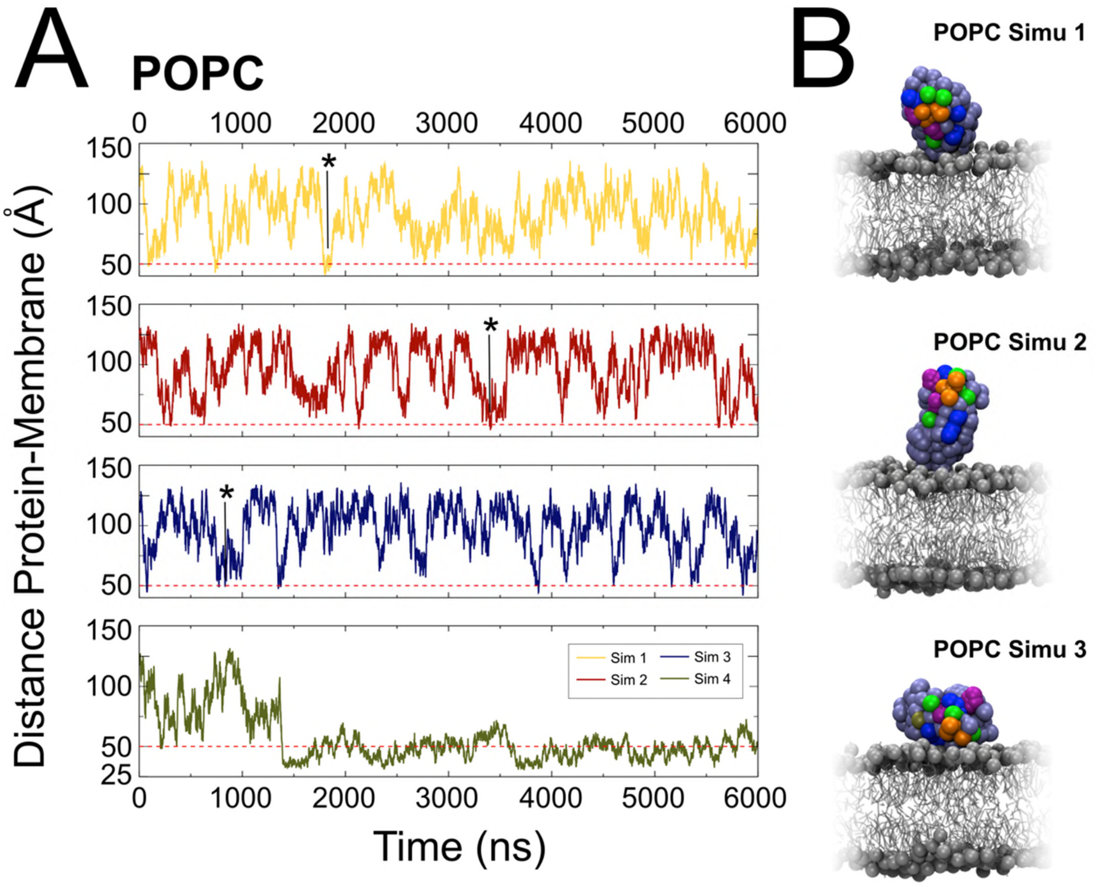

Figure S2 (related to Figure 1): distance between the FN2 domain and the membrane during CG simulations. (A) Evolution of the distance between the centres of mass of the membrane and of the FN2 domain in function of the time for CG simulations performed with the PC bilayer. (B) Different transient interactions of the FN2 domain with the PC membrane (highlighted by a star in A). Residues coloured in green, violet dark blue and orange are the main interacting residues depicted Fig. 2 A and S2. In (A) the dashed line depicts the distance from which the protein can touch the membrane, and in (B) Simu 1, Simu 2 and Simu 3 correspond to snapshots from the respective repeat simulations

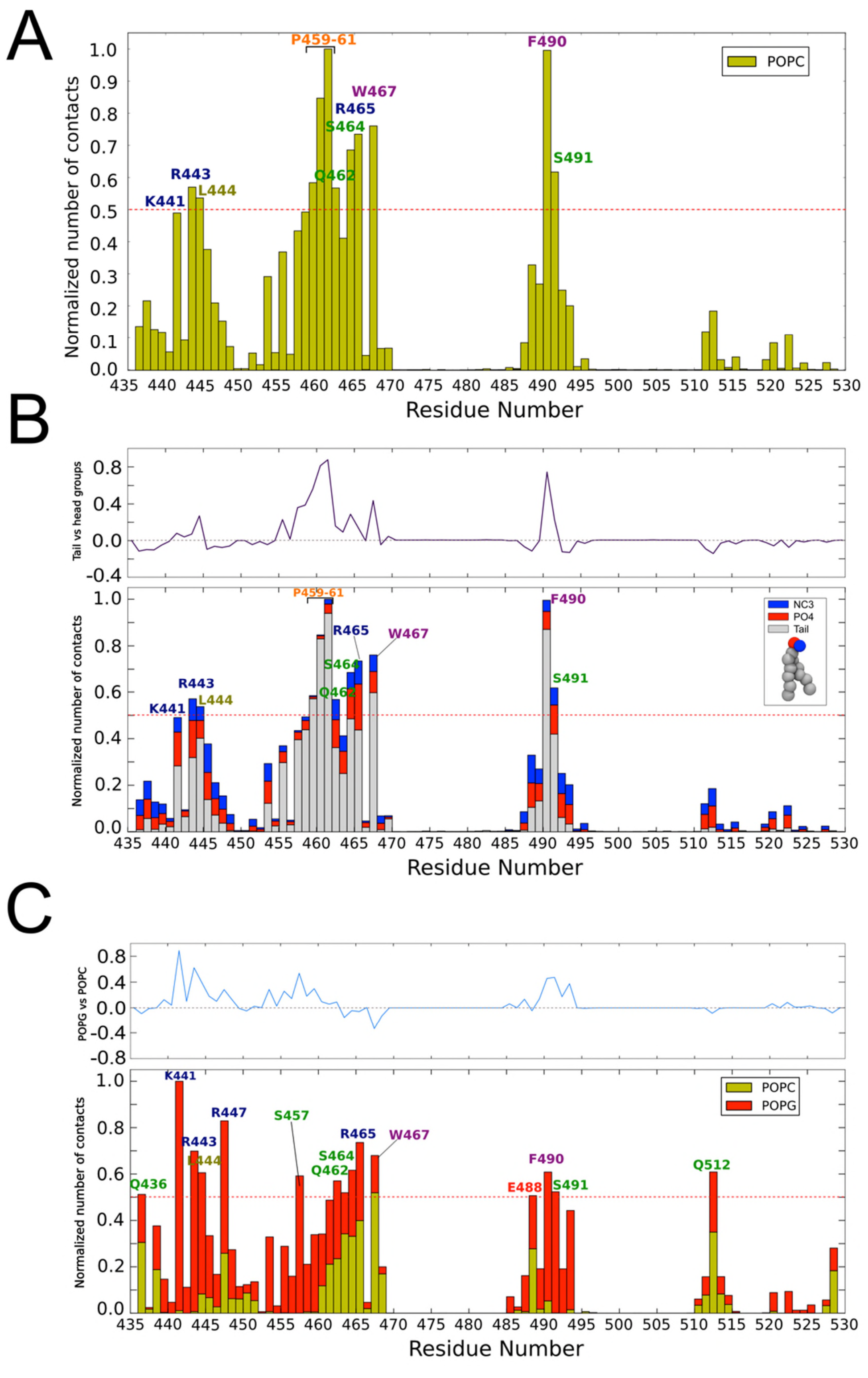

Figure S3 (related to Figure 1): Normalized averaged number of contacts. (A) Normalized average number of contacts of PC lipids as a function of residue number for the Wild Type. (B) Decomposition of the normalized average number of contacts for PC between NC3, PO4 particles and lipid tail. Dark curve displays the difference between the number of contact for the head group particles (NC3 and PO4) and the tail: positive values depict the preference of the residue for the tail. (C) Normalized average number of contacts of PC-PG membrane with the FN2 WT as a function of residue number for the AT simulations. The blue curve displays the difference between the number of contact for the PG lipids and PC lipids: positive values depict the preference of the residue for the PG lipids.

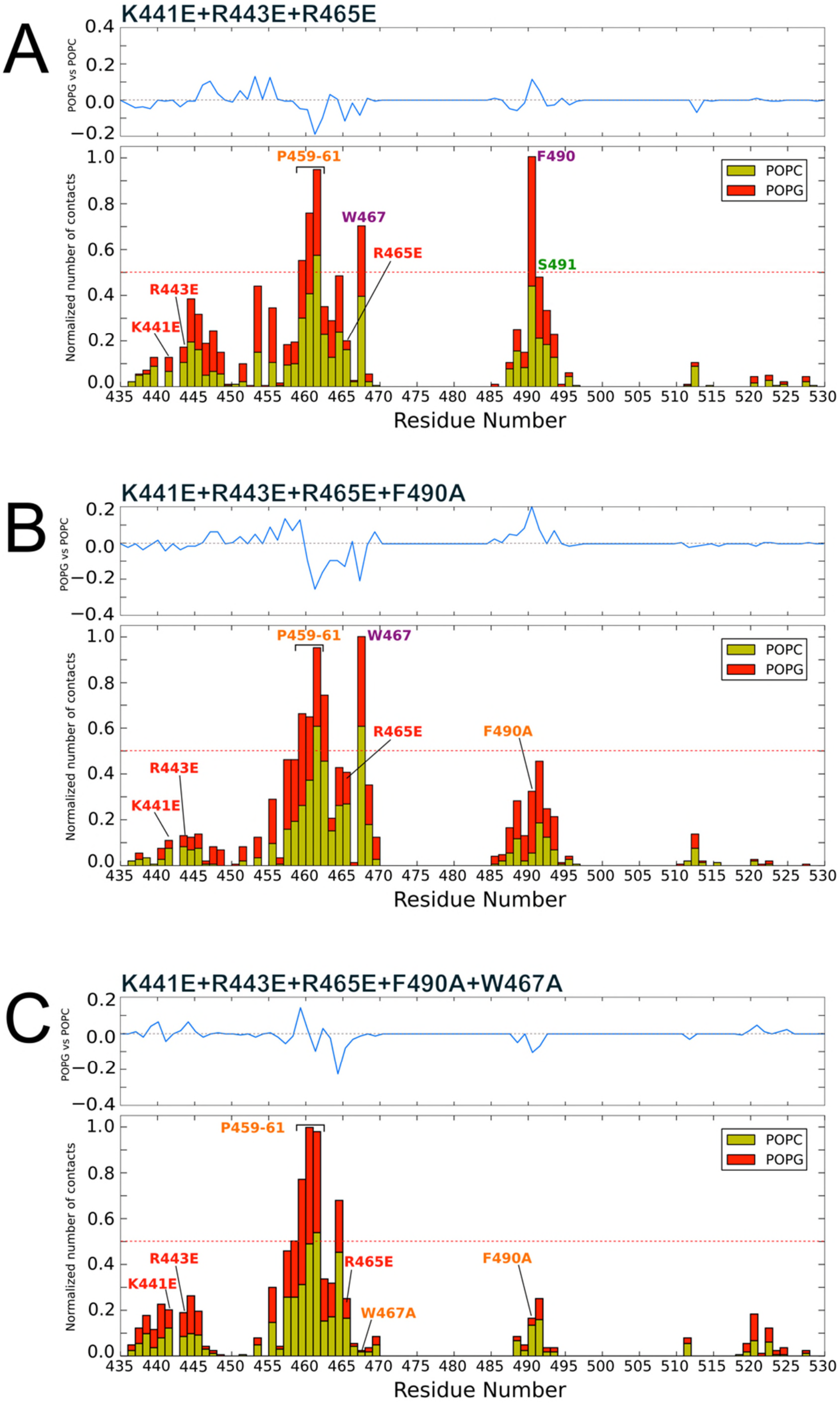

Figure S4 (related to Figure 2): Normalized averaged number of contacts for different mutants. Normalized average number of contacts of a PC-PG membrane with the (A) FN2 K441+R443+R365, (B) K441+R443+R365+F490A, and (C) K441+R443+R365 +F490A+W467A mutants as a function of residue number for the CG simulations. The blue curve displays the difference between the number of contact for the PG lipids and PC lipids: positive values depict the preference of the residue for the PG lipids.

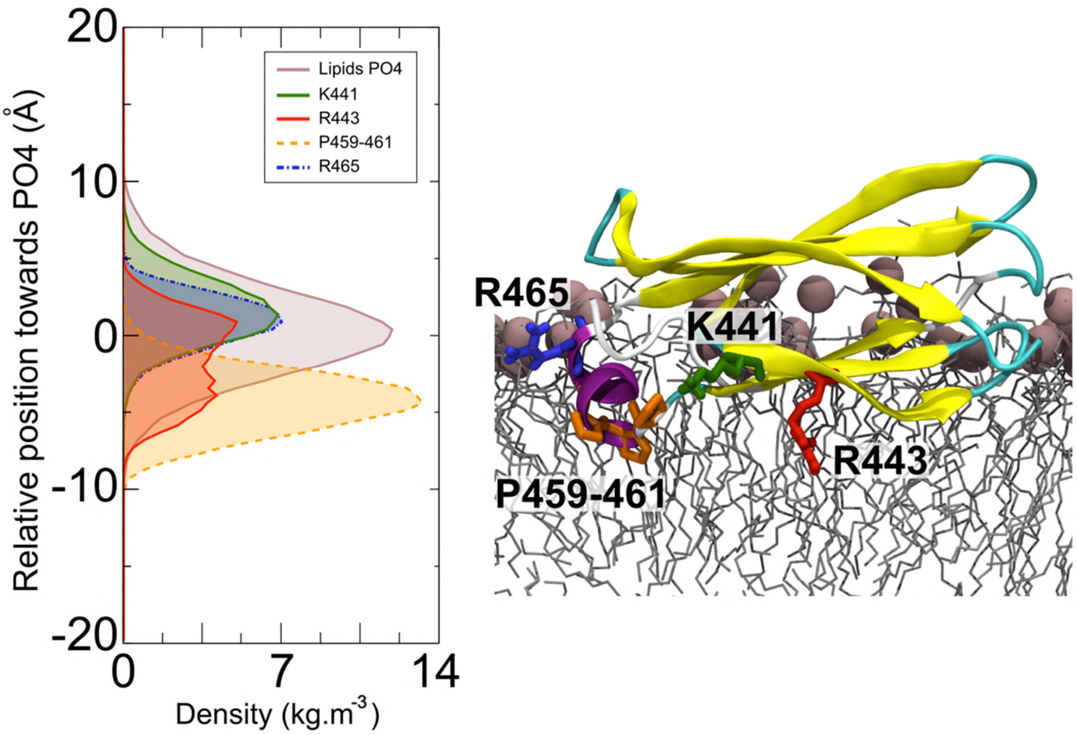

Figure S5 (related to Figure 4): FN2 positioning at the membrane. On left, the graph depicts the density profiles along the bilayer normal of several important residues for the interaction with the membrane. The profile is centred on the PO4 headgroup density. The amplitude of PO4 curve was divided by ten to fit in the graph. On right, a snapshot taken at the end of the AT simulation illustrates the graph results.

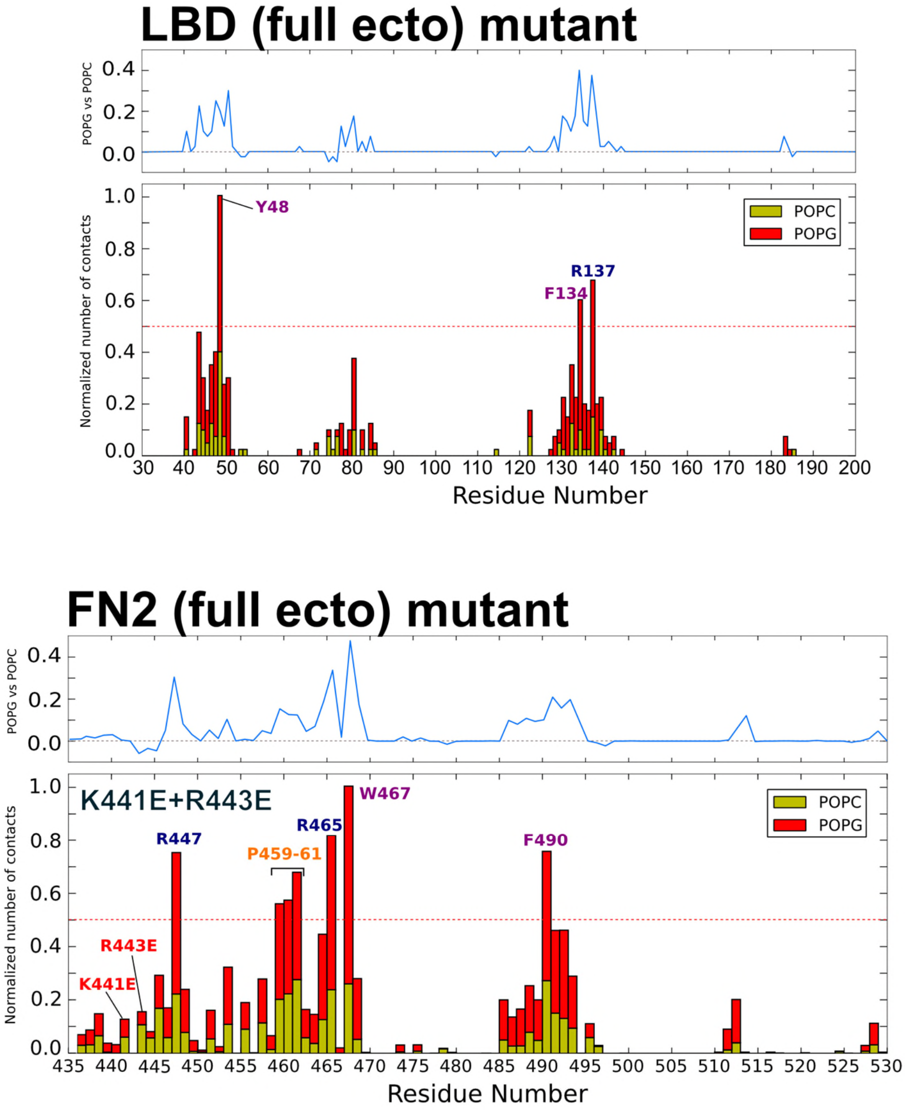

Figure S6 (related to Figure 6): Normalized averaged number of contacts of the LBD (upper panel) and FN2 domains (lower panel) with a PC-PG membrane for the K441E+R443E full ectododmain mutant. The blue curve displays the difference between the number of contact for the PG lipids and PC lipids: positive values depict the preference of the residue for the PG lipids.

3D Molecular Model (related to Figure 4):

A model of the FN2 domain at the membrane surface linked to the TM domain. Note that to obtain the numbering presented in the paper one has to add 435 to the residue number. This model is presented in Figure 4A of the paper.
