# Supplementary material for: Structures of the EphA2 Receptor at the Membrane: Role of Lipid Interactions
Source: Structure. 2016 Feb 2;24(2):337–47. doi: 10.1016/j.str.2015.11.008 (PMC4744086; doi:10.1016/j.str.2015.11.008)
Supplement: Document S2. Article plus Supplemental Information [file mmc3.pdf]

Theory

Structure

Structures of the EphA2 Receptor at the Membrane: Role of Lipid Interactions

Graphical Abstract

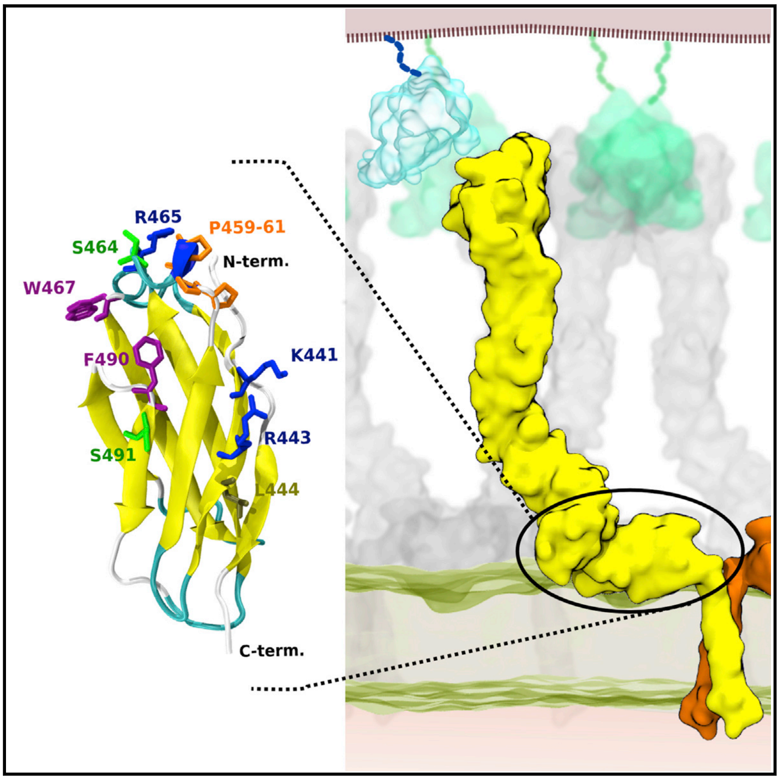

Authors

Matthieu Chavent, Elena Seiradake, E. Yvonne Jones, Mark S.P. Sansom

Correspondence

mark.sansom@bioch.ox.ac.uk

In Brief

Chavent et al. combine multiscale molecular dynamics simulations with a biochemical assay to describe the interactions of the EphA2 receptor FN2 domain with the anionic lipids of a model membrane. This provides insights into the dynamic behavior of the receptor at the cell surface.

Highlights

- EphA2 receptor ectodomain mainly interacts with lipid bilayers via its FN2 domain
- Anionic lipids are recruited by positively charged residues in FN2
- Mutations K441E and R443E abolish the specific binding of FN2 to anionic lipids
- The FN2-bilayer interaction orients the EphA2 ectodomain relative to a membrane

Chavent et al., 2016, Structure *24*, 337–347
February 2, 2016 ©2016 The Authors
http://dx.doi.org/10.1016/j.str.2015.11.008

CellPress

Structures of the EphA2 Receptor at the Membrane: Role of Lipid Interactions

Matthieu Chavent,[1] Elena Seiradake,[1,2] E. Yvonne Jones,[2] and Mark S.P. Sansom[1,*]
[1]Department of Biochemistry, University of Oxford, South Parks Road, Oxford OX1 3QU, UK
[2]Division of Structural Biology, Wellcome Trust Centre for Human Genetics, University of Oxford, Roosevelt Drive, Oxford OX3 7BN, UK
*Correspondence: mark.sansom@bioch.ox.ac.uk
http://dx.doi.org/10.1016/j.str.2015.11.008
This is an open access article under the CC BY license (http://creativecommons.org/licenses/by/4.0/).

SUMMARY

Ephs are transmembrane receptors that mediate cell-cell signaling. The N-terminal ectodomain binds ligands and enables receptor clustering, which activates the intracellular kinase. Relatively little is known about the function of the membrane-proximal fibronectin domain 2 (FN2) of the ectodomain. Multiscale molecular dynamics simulations reveal that FN2 interacts with lipid bilayers via a site comprising K441, R443, R465, Q462, S464, S491, W467, F490, and P459–461. FN2 preferentially binds anionic lipids, a preference that is reduced in the mutant K441E + R443E. We confirm these results by measuring the binding of wild-type and mutant FN2 domains to lipid vesicles. In simulations of the complete EphA2 ectodomain plus the transmembrane region, we show that FN2 anchors the otherwise flexible ectodomain at the surface of the bilayer. Altogether, our data suggest that FN2 serves a dual function of interacting with anionic lipids and constraining the structure of the EphA2 ectodomain to adopt membrane-proximal configurations.

INTRODUCTION

The ephrin receptors (Ephs) are the largest group within the family of receptor tyrosine kinases (RTKs). Ephs play critical roles in many developmental processes (Herbert and Stainier, 2011; Lai and Ip, 2009) and are implicated in a number of cancers (Herbert and Stainier, 2011; Lai and Ip, 2009; Pasquale, 2010). Ephs are grouped into two classes, A and B. Class A Ephs bind preferentially to ephrin A ligands, which are membrane-tethered through a glycosylphosphatidylinositol anchor. Class B Ephs preferentially bind ephrin Bs, which are attached to the membrane via a transmembrane (TM) helix (Kullander and Klein, 2002). Cross-interactions can occur between Eph A receptors and ephrin B ligands and vice versa (Bowden et al., 2009; Himanen et al., 2004; Qin et al., 2010). All Eph receptors share a common domain architecture (Figure 1A). The ectodomain is made up of a ligand-binding domain (LBD), which interacts with ephrin ligands, a Sushi domain, an epidermal growth factor-like (EGF) domain, and two fibronectin type III domains (FN1 and FN2). Thus, FN2 is the most membrane-proximal subdomain of the ectodomain. The intracellular region contains a tyrosine kinase domain, a sterile α-motif domain, and, sometimes, a PDZ-binding motif. A single TM helix, flanked by juxtamembrane linkers, connects the ectodomain and the intracellular region. Recent crystal structures of the entire ectodomains of two Eph receptors, EphA2 and EphA4, in complex with and without ephrin ligands, have revealed that ligand-induced Eph clustering is driven to a large extent by the N-terminal LBD and Sushi domains (Himanen et al., 2010; Seiradake et al., 2010, 2013; Xu et al., 2013).

Although detailed structural data are now available for all intra- and extracellular Eph domains (Davis et al., 2008; Himanen, 2011; Lee et al., 2012; Stapleton et al., 1999; Wiesner et al., 2006; Wybenga-Groot et al., 2001), and models of the TM helix dimer have been developed (Bocharov et al., 2008, 2010; Chavent et al., 2014; Sun et al., 2015), it remains poorly understood how the receptor is oriented relative to the lipid bilayer component of the cell membrane. An intriguing feature found in all crystal structures of the EphA2 ectodomain solved to date is that the membrane-proximal FN2 domain is oriented at an angle to the remainder of the ectodomain (Himanen et al., 2010; Seiradake et al., 2010). This suggests that the FN2 domain could lie on the cell membrane "sideways on," possibly presenting an extended interaction surface to the headgroups of the lipid molecules via one of its β sheets. Significantly, both computational (Arkhipov et al., 2013, 2014; Franco-Gonzalez et al., 2014; Kaszuba et al., 2015; Kästner et al., 2009) and experimental (Roberts et al., 2012) studies suggest that other RTKs may interact with lipid bilayers via their ectodomains.

We have explored the interaction of the EphA2 FN2 domain with model lipid bilayers. Molecular dynamics (MD) simulations provide a powerful computational tool to explore the interactions between proteins and membranes (Biggin and Bond, 2015; Li et al., 2015; Stansfeld and Sansom, 2011b). This approach was previously used to define the interactions of peripheral membrane proteins and domains (e.g. PH [Lumb and Sansom, 2012], C2 [Jaud et al., 2007], PTEN [Kalli et al., 2013, 2014], and Talin [Kalli et al., 2010]) at the surface of model membranes, yielding results that are in good agreement with biophysical and mutational data. MD simulations have also been used to model adsorption of the FN^{III}9 domain of fibronectin on (non-biological) surfaces (Kubiak-Ossowska et al., 2014). Here, we used multiscale MD simulations to define a potential membrane-binding motif on the surface of the EphA2 FN2 domain. We show that this membrane-binding motif includes positively charged residues that recruit negatively charged lipids to the site of membrane-protein interaction. Charge-swap mutations in the FN2

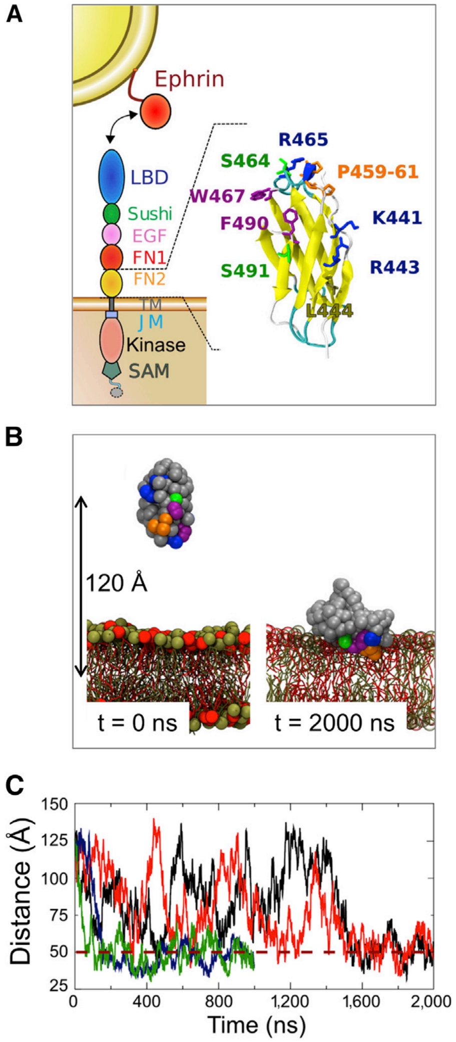

Figure 1. EphA Receptor

(A) Schematic diagram of an EphA receptor, showing its constituent domains and the interaction with an ephrin A ligand. The image on the right shows the structure of the membrane-proximal FN2 domain of the EphA2 receptor indicating key residues.

(B) Snapshots at the start and end of a CG simulation. At the end (t = 2000 ns), the FN2 domain interacts with the lipid bilayer headgroups (PC in tan; PG in red). The colors of the main residues interacting with the membrane correspond to those used in (A). See also Figure S1 for the setup of the coarse grain systems and the self-assembly protocol.

(C) Evolution of the distance between the center of mass of the bilayer and of the FN2 domain as a function of time for four CG simulations probing the interaction of the FN2 domain with a PC-PG bilayer. The dashed line indicates the approximate distance (50 Å center of domain to center of bilayer) when the protein is contacting the surface of the membrane.

See also Figures S2 and S3.

membrane-binding site reduce the recruitment of negatively charged lipids in silico, and abolish preferential binding to negatively charged lipids in a biophysical binding assay. We further demonstrate that the interactions of FN2 with lipids stabilize the otherwise flexible EphA2 ectodomain in two main conformations relative to the membrane. This was extended to simulations of two different models of the EphA2 dimer: one corresponding to a liganded conformation of the ectodomain, and one to an unliganded conformation. Taken together, our results reveal a previously unknown selective lipid-binding motif on the EphA2 ectodomain that defines the receptor's architecture at the membrane.

Table 1. Summary of the Simulations

System	Granularity	Particles	Duration (μs)
FN2 WT + PC bilayer	CG	17,063	4 × 6.0
FN2 WT + PC bilayer (self-assembly)	CG	9,575	4 × 2.5
FN2 WT + PC/PG bilayer	CG	17,407	2 × 1.0, 2 × 2.0
FN2 K441E + R443E + PC/PG bilayer	CG	17,430	1 × 1.0, 1 × 2.0, 2 × 3.0
FN2 K441 + R443E + R465E + PC/PG bilayer	CG	17,442	1 × 1.0, 1 × 2.0, 1 × 5.0, 1 × 6.0
FN2 K441 + R443E + R465E + F490A + PC/PG bilayer	CG	17,447	1 × 2.0, 1 × 4.0, 1 × 5.0, 2 × 6.0
FN2 K441 + R443E + R465E + F490A + W467A + PC/PG bilayer	CG	17,440	2 × 2.0, 2 × 7.0
Ectodomain ("flat") + TM + PC/PG bilayer	CG	196,851	1 × 10.0
Ectodomain ("upright") + TM + PC/PG bilayer	CG	295,445	1 × 10.0
Ectodomain dimer ("flat") + 2 TMs + PC/PG bilayer	CG	189,251	1 × 10.0
Ectodomain dimer ("upright") + 2 TMs + PC/PG bilayer	CG	292,430	1 × 10.0
FN2 WT + PC/PG bilayer	AT	89,156	1 × 300 ns
FN2+TM WT + PC/PG bilayer	AT	85,187	1 × 20 ns

Simulations were performed in mixed PC/PG or in PC bilayers. See also Table S1 for further details of FN2 domain interactions with the membrane.

RESULTS

Interaction of the FN2 Domain with Lipids

We performed coarse-grained (CG) dynamics simulations (summarized in Table 1 and Figure S1) with a PC (phosphatidylcholine) zwitterionic lipid bilayer, using a protein/bilayer encounter protocol (Kalli and Sansom, 2014; Kalli et al., 2010) to determine the preferred orientation and interaction of FN2 with a lipid bilayer. The initial position of the FN2 domain was such that the protein was distant (~120 Å) from the center of mass of the membrane (Figure 1B and Experimental Procedures). We performed multiple CG simulations, each lasting 6 μs, starting with different protein orientations with respect to the membrane. In three out of four simulations the protein interacted transiently with the membrane without adopting a stable orientation (Figure S2). In one simulation the FN2 domain bound to the membrane after ~1.4 μs. We also used a membrane self-assembly approach (Bond and Sansom, 2006; Bond et al., 2008) (see

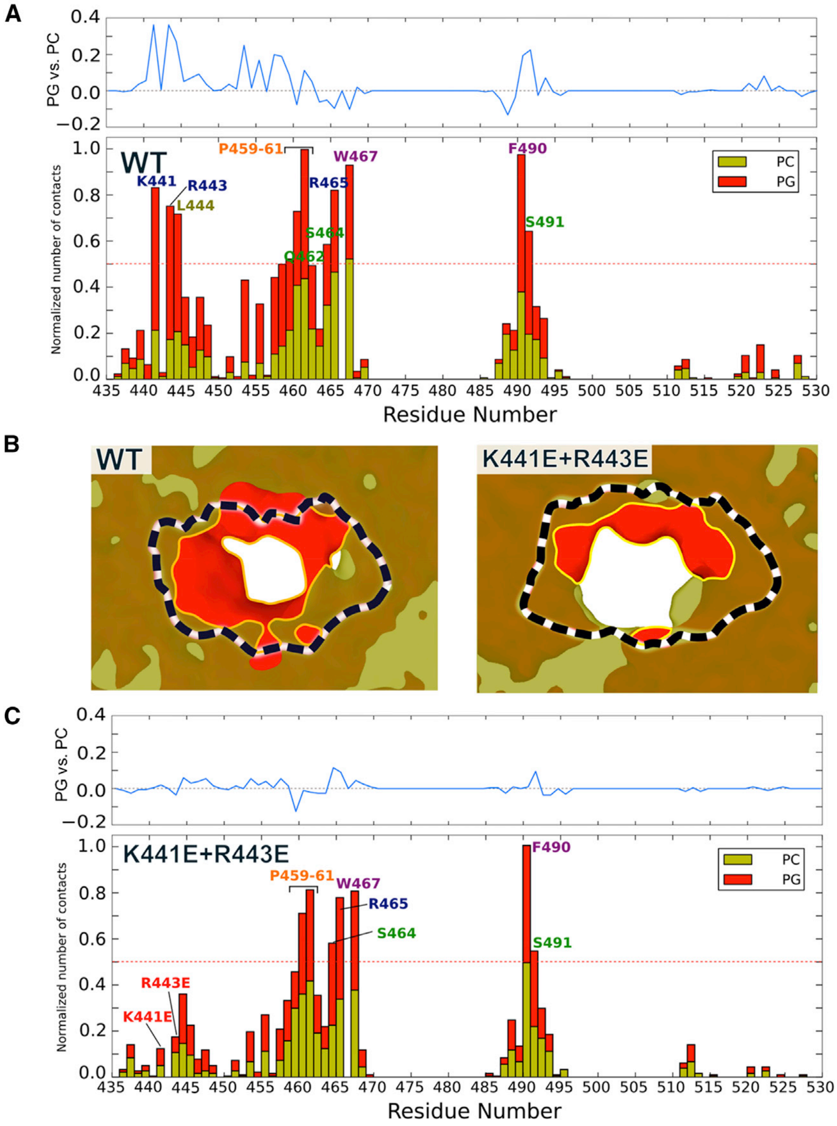

Figure 2. Interaction of FN2 Domain with a Bilayer

(A) The histogram shows the normalized average number of contacts (see Experimental Procedures for details) to the bilayer as a function of residue number for the wild-type (WT) FN2 domain. The blue curve above the histogram displays the difference between the number of contact for the anionic (PG) and zwitterionic (PC) lipid: positive values depict a preference of the residue for contacts to the anionic lipid headgroups.

(B) Occupancy density plots of lipid phosphate particles projected onto the bilayer plane. The red area indicates the presence of only anionic lipid headgroups, while the light-brown area represents the location of zwitterionic lipids and dark brown indicates the presence of both zwitterionic and anionic lipids. The black and white dashed line represents the FN2 domain. Plots are shown for the wild-type (WT) (left) and K441E + R443E mutant (right) domains.

(C) Normalized average number of contacts as a function of residue number for the K441E + R443E mutant.

See also Figure S4.

Experimental Procedures) to evaluate whether the membrane-bound form observed was the most stable arrangement of the FN2 domain at a bilayer surface. In four simulations, each lasting ~2.5 μs, we observed the same stable interaction between the FN2 domain and the lipid bilayer (Figure S1C). The main interacting FN2 residues are positively charged (K441, R443, and R465), polar (Q462, S464, and S491), or aromatic (W467 and F490). Three prolines (P459–461) reach deep into the membrane where they interact predominantly with the aliphatic lipid tails (Figures 1A, S3A, and S3B).

The presence of positively charged residues in the FN2-lipid interaction surface prompted us to also perform simulations using an anionic model membrane, thus mimicking anionic surfaces found in mammalian cell membranes. Negatively charged lipids such as the glycosphingolipid GM3 (Posse de Chaves and Sipione, 2010) are known to modulate RTK activity, for example that of the epidermal growth factor receptor (EGFR) (Coskun et al., 2011). However, such glycolipids exhibit rather complex behavior, including nanoscale clustering, in mixed lipid bilayers (Ingólfsson et al., 2014; Koldsø et al., 2014). We therefore decided to use, as a first approximation to the anionic membrane surface, a simpler lipid, namely phosphatidylglycerol (PG). We therefore constructed a model bilayer of 60% zwitterionic PC and 40% anionic PG (Figure S1B). With this anionic lipid bilayer, we observed stable interactions for all four of the simulations performed (Figures 1B and 1C). The FN2 surface interacting with the anionic bilayer was essentially the same as previously observed using neutral PC bilayers (Figure 2A). The N-terminal region of the FN2 membrane-binding motif contains a positively charged motif, including K441 and R443 (Figure 3A), which interacts preferentially with anionic lipids (Figure 2A). At the end of the simulation, the PG lipids formed a negatively charged "halo" around the bound FN2 domain (Figure 2B). This preference for anionic lipids was quantified by calculating radial distribution functions (RDFs) for the lipids around the protein (Figure 3B). This analysis yielded a ratio of the first RDF peak (corresponding to direct protein/lipid interactions) for PG/PC of 2.5:1. Given the composition of the bilayer, this result correspond to a ~4-fold binding preference of FN2 for anionic over neutral (i.e. zwitterionic) phospholipids. To refine our model, we converted the FN2 domain, as found in the last frame of the CG simulation, into an atomistic (AT) model (see Experimental Procedures). We simulated the resultant atomistic system for 0.3 μs. In this AT model, FN2 remained stably bound to the membrane without significant changes in orientation (Figure S5). Compared with the CG models, only minor differences were found, namely small increases in N-terminal interactions with charged residues alongside small decreases in interactions of apolar and aromatic residues (Figure S3C). We did not

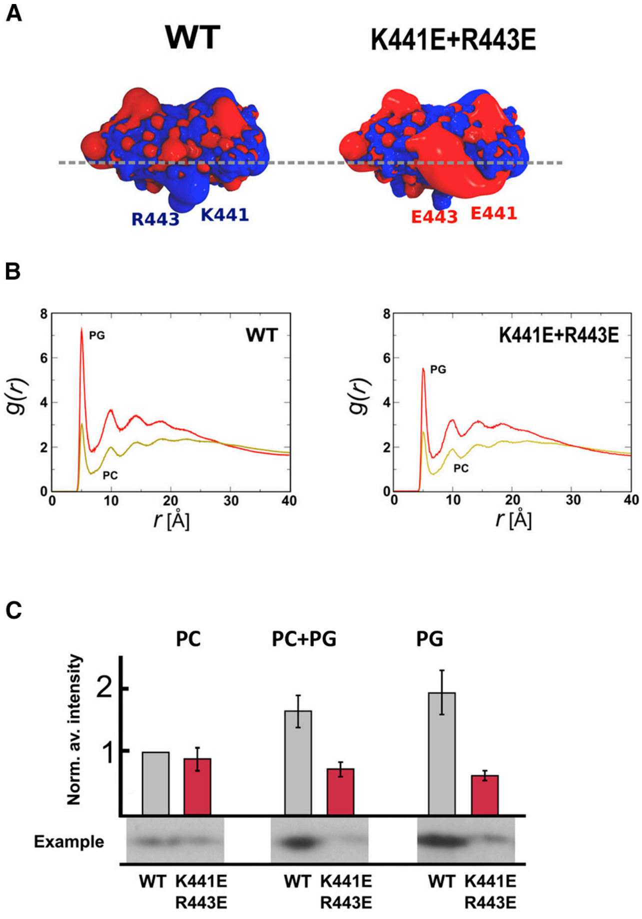

Figure 3. Preference of the FN2 Domain for Anionic Lipids

(A) The FN2 domain oriented to show its observed orientation when interacting with a membrane (lipid headgroup positions are depicted by a dashed gray line) for both the wild-type (WT) and the K441E + R443E mutant domain. The protein surface represents the electrostatic potential around the protein calculated with APBS (Baker, 2001).

(B) Radial distribution functions showing the distribution of anionic (PG; red) and zwitterion (PC; brown) lipid headgroup with respect to the protein. Each curve is the average of four simulations.

(C) Lipid vesicle pull-down experiment, showing the interaction of FN2 domains with PC, PC + PG, or PG lipids. Intensities measured on western blots were normalized using lane 1 (wild-type [WT] protein pelleted with PC) and averaged. In each set, we normalised the data using the corresponding value measured for wild-type protein pelleted with DOPC vesicles. After normalization, we calculated averages and SEM for all experiments.

observe differences in the density of PG lipids around the FN2 domain after 0.3 μs of simulation.

To validate our results experimentally, we performed lipid vesicle pull-down assays with isolated FN2 domain, expressed in and purified from HEK293T cells (Figure 3C). We used lipid vesicles composed of either PC or PG or of a 1:1 mixture of PC and PG. Consistent with the in silico results, we found that FN2-binding to these vesicles correlates with the ratio of PG/PC, with a preference for the negatively charged lipid PG. We note that there is no significant difference between PC + PG and PG liposomes. The anionic lipid-binding sites may be already fully saturated in the mixed PC + PG liposomes, such that no further increase is seen with the pure PG vesicles.

Effects of Mutations on the FN2 Preference for Anionic Lipids

Our simulation highlighted a strong spot of anionic lipid attraction around the residues K441 and R443 (see curve in Figure 2A). We mutated both residues to glutamic acids (K441E + R443E) to assess the contribution of charge effects on FN2-lipid binding specificity (Figure 3A). We performed four simulations with the mixed PC-PG bilayer. The simulations showed that mutant FN2 still interacts with the membrane via the same motif as with the wild-type domain, although the time taken to reach the membrane-bound orientation was longer (Table S1). Importantly, the mutations had an impact on the FN2 domain's preference for anionic lipids. RDFs revealed less anionic lipid around the mutant FN2 domain compared with the wild-type: *g(r)* for the first peak changes from 7.5 for the wild-type (Figure 3B) to 5.5 for the mutant. The density of PG lipids at the membrane-protein interface was lower for the mutant than for the wild-type (Figure 2B). Residues 440–450 also interacted less strongly with the membrane in the mutant compared with the wild-type (Figure 2C). In contrast, the K441E + R443E mutations had no significant effect on FN2 interaction with PC lipids. We extended the charge-swap mutations to include R465E (Figure S4A). The protein still interacted with the membrane, although for one repeat it did not form a stable interaction after 6 μs of simulations. The anionic lipid preference profile for this triple mutant (i.e. K441E + R443E + R465E) was very similar to that of the K441E + R443E mutant. We continued to add further mutants, adding F490A and W467A to the triple mutant (Figures S4B and S4C). Again, these mutants did not fully abolish the interaction with the membrane, but the time elapsed before an interaction was formed did increase slightly in comparison with the other mutants (Table S1). Thus, the clearest effect was for the double K441E + R443E mutant, which seems to almost fully abolish the preference for anionic lipids.

To validate our simulation results, we produced the double mutant protein FN2 K441E + R443E in vitro and performed vesicle pull-down assays. Unlike the wild-type, mutant FN2 protein did not bind better to vesicles of PG or of PC + PG compared with PC (Figure 3C). We conclude that the preferential interaction of FN2 with anionic lipids is dependent on the membrane-binding motif described above, containing K441 and R443.

Modeling EphA2 Signaling Units in a Lipid Bilayer Environment

The FN2 domain orientation at the membrane surface was then used to construct more complex assemblies, including monomers and dimers of EphA2 receptors (i.e. the ectodomain + TM helix) with the membrane, thus integrating structures from both crystallography (Seiradake et al., 2010) and nuclear magnetic resonance (NMR) (Bocharov et al., 2010) experiments.

We first constructed an atomistic model of the FN2 domain attached to the TM helix corresponding to the NMR structure of the TM dimer (Bocharov et al., 2010) (see Experimental Procedures). We performed a short (20 ns) AT simulation to relax the

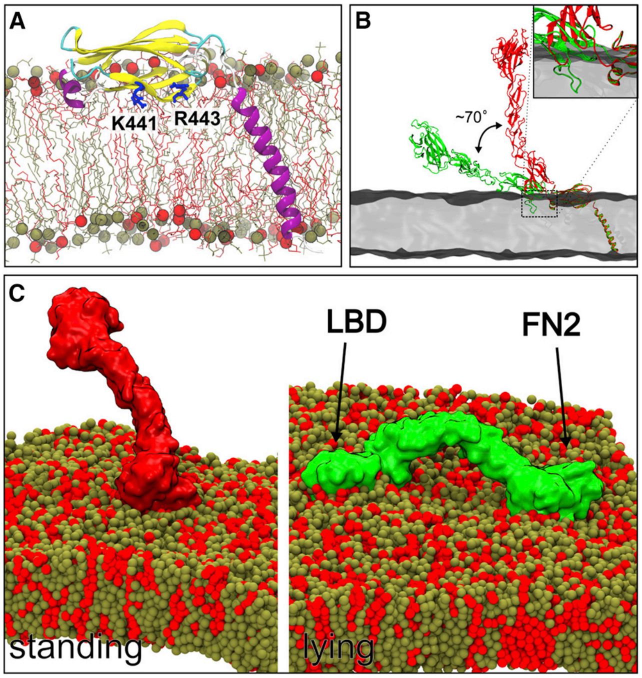

Figure 4. Integrative Model of the EphA2 FN2 Domain plus Transmembrane Helix Interacting with a Membrane

(A) All-atom model of the FN2 domain and the transmembrane domain at 20 ns during an AT MD simulation. The coordinates of the model of the FN2 and TM domains at the membrane are available in Supplemental Information. See also Figure S5.

(B) Models of liganded (red) and unliganded (green) state of the EphA2 ecto + TM domains in a bilayer (gray). The inset focuses on residues of the 367–375 loop of the FN1 domain relative to the membrane (in gray).

(C) Snapshots of two configurations of the ecotodomains at the end of 10-μs simulations. On the left, the configuration obtained starting from the ligand-bound state of the ectodomain stayed relatively orthogonal to the membrane, while starting from the ligand unbound state the simulation resulted in "collapse" of the ectodomain onto the membrane, such that the N-terminal LBD also interacted with the lipids.

resultant model (Figure 4A). The TM helix remained tilted in the membrane, as previously described for the dimeric TM domain structure (Bocharov et al., 2010). The linker remained close to the FN2 domain during the simulation. Residues L535, A536, and V537, which adopt a helical conformation in the NMR structure (Bocharov et al., 2010), had a tendency to unfold at the bilayer surface during our MD simulation, but the rest of the helix remained stable.

Based on this model, we then constructed two atomistic configurations of the monomer comprising the EphA2 ectodomain + TM helix embedded in the lipid bilayer. This corresponds to a construct that, in vitro, is functionally capable of clustering at the cell surface in response to ligand stimulation (E.S., unpublished data). We generated models based on the liganded conformation (PDB: 2X11; but without the ephrin ligand) and on the unliganded (PDB: 2X10) conformation (see also Experimental Procedures). It was previously reported that the FN1-FN2 linker can undergo substantial conformational changes, leading to different structures for the FN1-FN2 domains with the FN2 domain rotated by ~70° between the two models (Seiradake et al., 2010). The rest of the ectodomain (i.e. the LBD to EGF domain) appears to be less flexible. Here, the positioning of the FN2 domain onto the membrane surface as directed by our simulations provided a novel insight into the potential consequences of this structural difference for receptor orientation at the cell surface. The anchoring of the FN2 domain to the membrane surface yields two very different orientations of the receptor as a whole driven by the FN1-FN2 linker difference. The unliganded configuration lies almost flat on the membrane surface, while the ephrin-bound conformation adopts a more upright orientation (Figure 4B). Neither orientation leads to major steric clashes of the ectodomain with the membrane, although loop residues 367–375 of the FN1 domain, which were not ordered in the original structure, may point toward and form close contacts with the membrane (Figure 4B, inset).

We used the monomeric EphA2 ecto + TM models described above to initiate two CG simulations (each of 10 μs duration; see Figure 4C and Experimental Procedures). The model in which the ectodomain is lying "flat" on the surface of the membrane (derived from the unliganded EphA2 ectodomain structure, PDB: 2X10) resumed a stable position in which both the FN2 domain and the LBD domain interact with the membrane. The LBD residues involved in this interaction were largely aromatic. Both the FN2 and LBD domain interactions attract anionic lipids (Figure 6A). The ephrin ligand-binding site remains accessible in this conformation, i.e. it faces upward, away from the membrane (Figure 6B). During the 10-μs simulation of the "upright" model (derived from the ephrin-bound EphA2 ectodomain structure, PDB: 2X11, albeit without the ligand present during the simulation), the flexible FN1-FN2 linker allowed for movement of the EphA2 ectodomain, although the receptor remained in a generally upright orientation (Figures 4C and 5A). The distance between the LBD domain and the membrane ranged from 75 to 150 Å (Figure 5B). As previously shown for the FN2 domain alone, the RDF calculation for the two ectodomain configurations highlighted a preference of anionic lipid around the FN2 domain (data not shown). We then simulated a K441E + R443E mutant, as previously done for the FN2 domain only, also in the context of the entire ectodomain in the upright conformation. Interestingly, this mutant is destabilized in comparison with the wild-type, and after 6 μs the ectodomain lies flat on the membrane, resembling the configuration of the unliganded structure (Figure 5C). Following this change in orientation, the interactions of this mutant with the membrane are very similar to those of the unliganded structure (Figure S6). Taken together, our data suggest that, while the FN2 domain is constrained by tight interactions with the lipid bilayer surface, flexible regions upstream of FN2 allow for a range of EphA2 ectodomain orientations relative to bilayer/membrane.

We extended our study of the monomeric receptor by simulation of crystal-structure-based dimers of the liganded and unliganded forms (see Experimental Procedures). We note that, as for the monomeric models, the creation of the dimers did not lead to any major steric clashes with the membrane. After 10 μs of simulations we noted dynamic differences when compared with the monomer simulations. For the upright dimer the configuration stayed very stable during the whole simulation, unable to adopt a conformation as flexible as the monomer

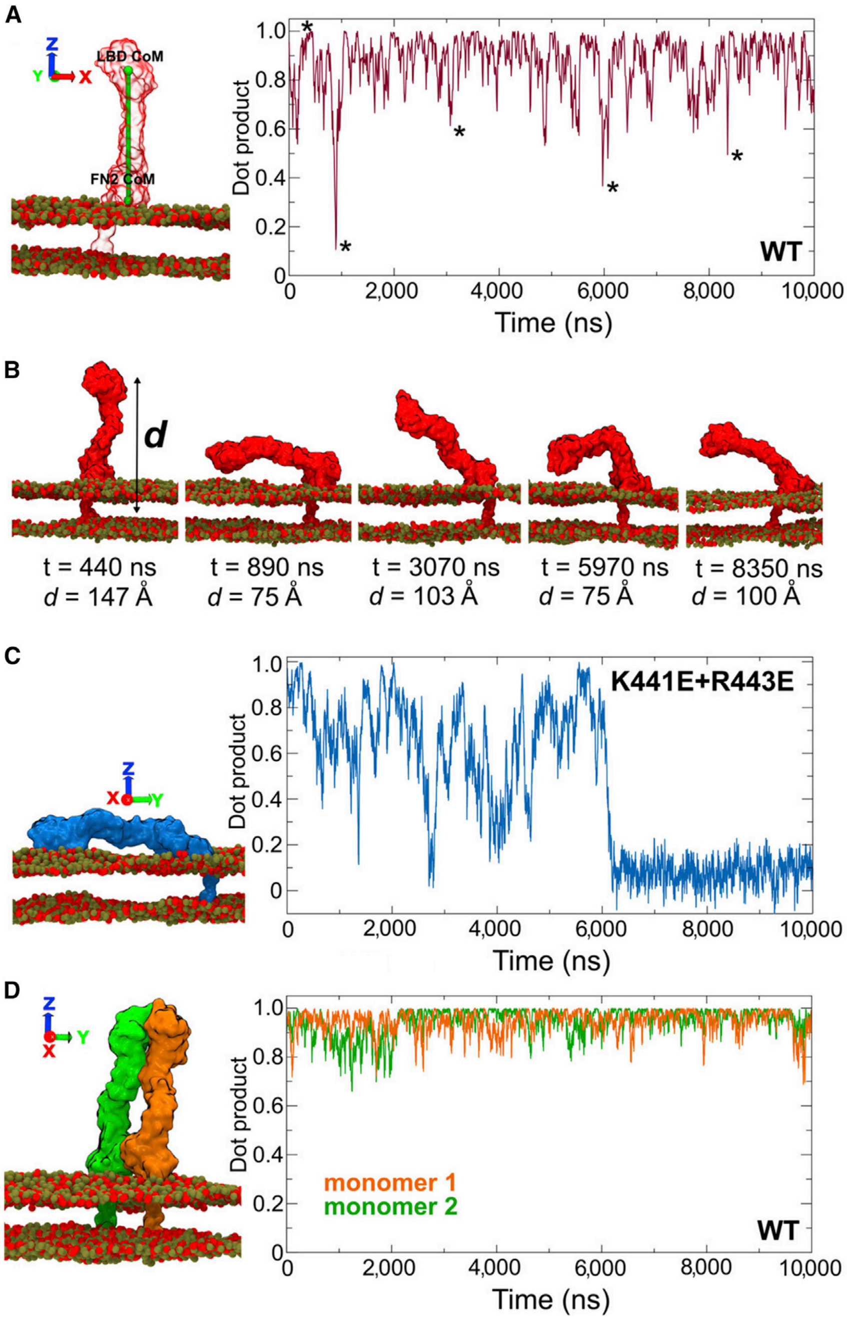

Figure 5. Flexibility in the Liganded EphA2 Monomer and Dimer Ectodomain + TM Domain Systems

(A and B) Flexibility of the wild-type monomer (WT and K441E + R443E mutant) (A) showing that while for the majority of the time the ectodomain is approximately orthogonal to the membrane, there is a degree of flexibility. Stars depict bent or lying-on-the-membrane orientations of the ectodomain, as presented in (B), which also provides the distance (*d*) of the LBD from the center of the bilayer.

(C) Flexibility of the K441E + R443E monomer and a snapshot at the end of the simulation.

(D) Flexibility of the WT dimer system. To evaluate ectodomain flexibility in our simulations, we calculated the dot product between the z vector (i.e. perpendicular to the membrane) and a vector formed by the centers of mass of the LBD and FN2. The dot product is close to 1 when the ectodomain is orthogonal to the membrane and close to 0 when the ectodomain lies flat on the surface of the membrane.

(Figure 5D). For the unliganded dimer, as was seen for the monomer, the ectodomains quickly (within a few tens of nanoseconds) moved down onto the bilayer surface where they remained for the whole 10 μs. One monomer is positioned on top of the other, resulting in asymmetrical interactions with the membrane (Figure 6C). The first monomer presents interactions equivalent to those seen in the monomeric simulations, interacting mainly with the membrane through the FN2 domain and LBD (Figure 6D). The asymmetry is also visible in terms of lipid preference between the two monomers. Interestingly, asymmetric receptor dimers are suggested to occur in other signaling systems, such as the EGFRs (Arkhipov et al., 2014).

DISCUSSION

We have presented MD simulations and biophysical data showing that the EphA2 ectodomain harbors a membrane-binding motif in the FN2 domain which preferentially interacts with anionic lipids. The cell membrane is composed of different lipids, often arranged into specific domains, whose biological functions are only beginning to emerge. For example, lipid (nano) domains (sometimes denoted as "lipid rafts") are densely packed regions that are enriched in signaling proteins and play important roles in membrane signaling events (Simons and Toomre, 2000). The lipid composition of these nanodomains differs from the average, overall neutral, composition of the mammalian cell membrane (van Meer et al., 2008). Nanodomains are often enriched in anionic lipids (Pike et al., 2002), such as glycolipid GM3 (Koldsø et al., 2014). Activated EphA2 is known to localize to lipid nanodomains (Chakraborty et al., 2012) and requires the presence of negatively charged lipids for at least some of its functions (Tawadros et al., 2012). As demonstrated previously for the EGFR (Hofman et al., 2008), interaction of EphA2 with specific anionic lipids could act as a mechanism to trigger the coalescence of lipid nanodomains to form more extended signaling platforms. Conversely, the clustering of anionic lipids around EphA2 could guide the receptor toward specific lipid domains, in agreement with the lipid shells theory (Anderson and Jacobson, 2002).

The simulations of the EphA2 ecto + TM domains suggest that the receptor adopts at least two different conformations at the cell surface. In one arrangement the receptor ectodomain sits "upright" on the membrane while in the second it lies "flat" on

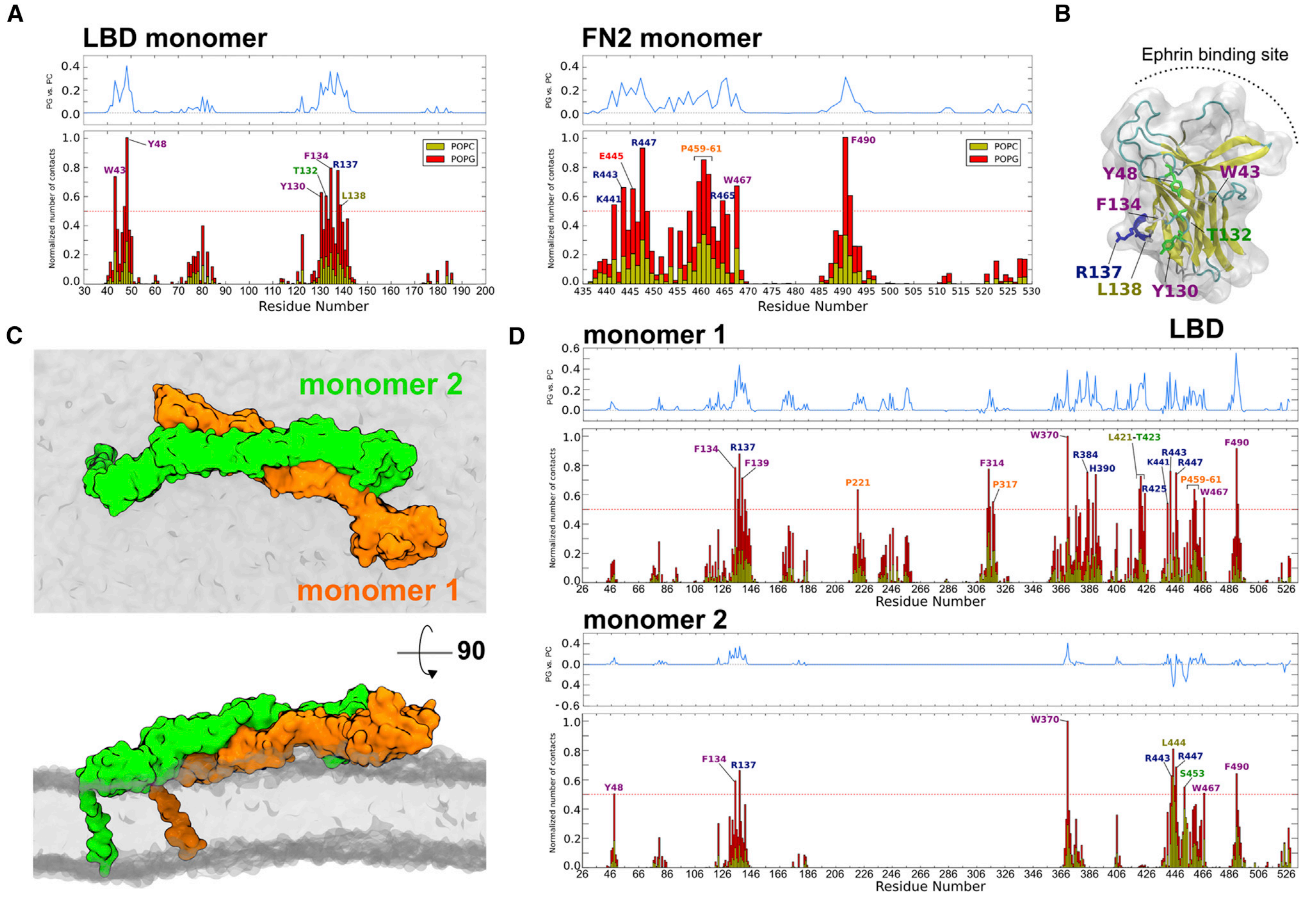

Figure 6. EphA2 Ectodomain Interacting with the Membrane Starting from the Unliganded Conformation

(A) Histogram showing the normalized average number of contacts (see Experimental Procedures for details) to the bilayer as a function of residue number for the LBD and FN2 domain, which are interacting with the membrane (Figure 4C).

(B) Main residues of the LBD domain interacting with the membrane. The ephrin-binding site is not involved.

(C) A snapshot of the unliganded dimer at the end of the simulation showing the asymmetric interaction of the two monomers with the membrane.

(D) Histogram of the normalized average number of contacts for the two monomers starting from the unliganded dimer conformation.

See also Figure S6.

the membrane surface. Both conformations are consistent with known biological receptor functions. In the upright conformation the receptor LBD is in a position presumably compatible with binding ephrin ligand presented by a neighboring cell (Figure 7). This conformation also allows packing of the receptor into dense signaling clusters as previously proposed based on crystal structures (Himanen et al., 2010; Seiradake et al., 2010). Our simulations suggest that the FN2-membrane interaction we describe here has a stabilizing effect on this EphA2 conformation, which can be removed by mutation of the relevant membrane-binding site on FN2. The flat orientation of the EphA2 ectodomain on the membrane surface could represent a functionally distinct receptor conformation, as postulated for EphA4 based on a recent crystal structure data, and seems to also exist for the EphA2 receptor, perhaps in a less stable conformation due to a smaller interface between the FN2 and LBD domains (Xu et al., 2013). As recently suggested by Nikolov et al. (2014), the two EphA2 conformations we describe may reflect clustering at different receptor densities, with the "flat" configuration corresponding to the formation of less tightly packed clusters compared with the "upright" conformation. Cluster density may in turn have an effect on the signaling outcome (Lohmüller et al., 2013). Xu et al. (2013) have postulated that the "flat" configuration may also help to create a larger platform to propagate ephrin-Eph signaling. Nevertheless, as seen for other RTKs, such as EGFR, it is still not clear how membrane lipid composition may favor active or inactive forms (Arkhipov et al., 2013, 2014; Kaszuba et al., 2015; Kästner et al., 2009; Webb et al., 2008). Furthermore, the membrane environment (Arish et al., 2015) and/or the glycosylation of the protein (Kaszuba et al., 2015) may impact on the behavior of RTKs. Interestingly, the "flat" EphA2 conformation appears to be compatible with ephrin ligand binding, as the main ephrin-binding site is not obscured. This conformation could, for example, promote in *cis* interaction with ephrins, also found in lipid nanodomains (Gauthier and Robbins, 2003; Levental et al., 2010). This would be in addition to a second in *cis* ephrin-binding site thought to exist at the Eph FN domains (Carvalho et al., 2006; Falivelli

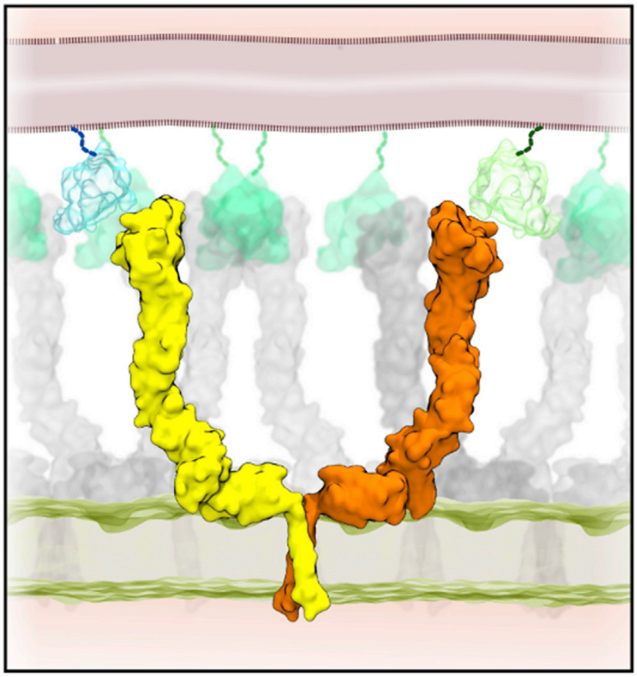

Figure 7. EphaA2 Receptor Clustering

An integrated model (based on NMR and X-ray structural data in combination with our "upright" simulation model of the ecto + TM domain in a bilayer) of the structure of an Epha2 dimer is used to produce an illustrative model of EphaA2 receptor clustering. In the center (in yellow and orange) a dimer of receptor interacting through the TM helices (PDB: 2K9Y) is shown, combined with (in gray) the X-ray structures of an array of EphA2 receptors (Seiradake et al., 2010) (PDB: 2X11). Ephrin ligands are shown in green and blue along with a schematic representation of the bilayer of an opposing cell membrane (broken wavy lines).

et al., 2013). Thus, the data presented here form the basis for future work exploring in *cis* interactions of Eph receptors with ephrin and other cell-surface molecules.

Eph receptors are known to dimerize and further cluster upon ligand-induced activation. Our simulations show that the upright orientation seemed to be less flexible than for the monomeric receptor, a characteristic that may help to retain a favorable conformation to propagate formation of a signaling array by addition of monomers (Seiradake et al., 2010). Singh et al. (2015) proposed recently that the formation of unliganded dimers might reduce EphA2 pro-tumorigenic signaling by reducing the supply of free monomers. In our simulations, we can see that the unliganded dimer configuration might sequester monomeric forms at the membrane and potentially hide (by interactions with the membrane surface) important binding sites for the seeding effect (see Seiradake et al., 2010) including residues 246–248, 254–255–257, or 378 and 380 (Figure 6D, monomer 1). In our model, the distance between the two TM helices is ~52 Å, which is in agreement with a separation of ~48 Å from fluorescence resonance energy transfer measurements (Singh et al., 2015). We postulate that this configuration may impose some steric constraints: on the extracellular LBD side the extension of the signaling platform suggested by Xu et al. (2013) might be prevented; and on the cytosolic side of the membrane the kinase domains might be held apart, preventing them from signaling (Figure 6D).

The CG simulation protocol uses an elastic network model to maintain the tertiary structure of the proteins (Bond et al., 2007; Periole et al., 2009). This may dampen the larger-scale dynamic of the systems, so that in the future extended atomistic simulations could be used to further validate our model. Furthermore, the use of a CG force field might be anticipated to restrict the spatial and chemical resolution of protein/lipid contacts (Marrink and Tieleman, 2013). It is encouraging that we obtain a good correlation between simulation and experimental studies of the anionic lipid-binding preference. Our results using this PC/PG system will act as a stepping stone toward constructing more biologically relevant models using multicomponent lipid mixtures that better mimic the compositional complexity of living cell membranes (Ingólfsson et al., 2014) as well as the study of glycosylation effects (as in recent studies of EGFR [Kaszuba et al., 2015]).

Taken together, the biophysical and computational data presented here increase our molecular understanding of how EphA2 receptors are oriented and function within a model lipid membrane. Significantly, the model presented provides an essential stepping stone toward the analysis of full-length Eph receptor structure at the cell surface, its clustering (Figure 7), and the mechanism of how Eph receptors transfer signals across the cell membrane.

EXPERIMENTAL PROCEDURES

System Construction

FN2 Domain Interacting with the Membrane

We used two different protocols. We coarse grained the FN2 domain (Figure S1A) using an in-house protocol. An elastic network was applied to further constrain the tertiary structure of the protein, using a cut-off of 7 Å. We then positioned the domain ~120 Å away from the center of mass of the membrane (Figure 1B) rotating it about the x, y, or z axes to create four different starting orientations (Table S1). We used this protocol to assess the interaction of the FN2 domain with a palmitoyloleoylphosphatidylcholine (POPC) membrane, and with a mixed POPC/palmitoyloleoylphosphatidylglycerol (POPG) (3:2) membrane. We also used the four different starting positions of the FN2 domain in a self-assembly approach whereby POPC lipids were initially in random positions around the protein. We identified the most representative structure of the FN2 domain interacting with the mixed PC/PG bilayer and converted the system to an atomistic representation using a CG2AT protocol, which has been used for a number of systems (Stansfeld and Sansom, 2011a). We then extracted one TM helix from the NMR structure of the EphA2 TM dimer (PDB: 2K9Y) (Bocharov et al., 2010) to generate an AT model of EphA2 FN2 fused to its TM helix in a PC/PG lipid bilayer. We modeled the seven-residue extracellular juxtamembrane linker (sequence: S528–G533) between FN2 and TM domains using Modeller (Eswar et al., 2007). We then applied the Alchembed method (Jefferys et al., 2015) to smoothly embed the FN2 domain + TM helix in the bilayer.

Ectodomain Monomers Interacting with the Membrane

We constructed two different systems, corresponding to the ectodomain monomers in an unliganded or liganded state using, respectively, one monomer from the 2X10 or 2X11 PDB entries. In the latter case, we removed the ephrin ligand. For each structure we used Modeller (Eswar et al., 2007) to construct missing loops. In each case we then superposed the full EphA2 ectodomain structure onto our atomistic FN2 domain model attached to the TM helix. We then concatenated the ectodomain part (i.e. LBD to FN1 domains) to our model FN2 + TM in the membrane. We then extended the lipid bilayer to create a large 4 × 4 patch of membrane. The resultant model was then converted to coarse grain using an in-house script. We used the VMD plugin mutator (http://www.ks.uiuc.edu/Research/vmd/plugins/mutator/) on

the atomistic model of the liganded monomer to construct the K441E + R443 mutant, then coarse grained it as described above.

Ectodomain Dimers Interacting with the Membrane

Based on the atomistic monomer models, we constructed each dimer by superimposing each of two ectodomain monomers onto the PDB structures of the ectodomain + TM dimers, then placing the full dimer in the membrane. We coarse grained the membrane and the proteins (making sure that we did not link the monomers together by the elastic network) as described above. We then applied the Alchembed methodology (Jefferys et al., 2015) to remove clashes due to the insertion of the new monomer in the membrane.

CG MD Simulations

CG MD simulations were performed using GROMACS versions 4.5. and 4.6 (www.gromacs.org) (Hess et al., 2008) and the MARTINI 2.1 force field (Monticelli et al., 2008). For the PC membrane, a pre-assembled membrane composed of 249 POPC molecules was used for the encounter protocol while the self-assembly method involved 242 lipids. Water and counterions were added to equilibrate the system. For the mixed PC/PG bilayer, we used a pre-assembled bilayer from previous work (Kalli et al., 2010) composed of 151 POPC and 98 POPG lipids. After 100 steps of steepest descent, we performed 5 ns of equilibration before the production runs (see Tables 1 and S1 for details). In the case of the self-assembly method, after the 500 ns of self-assembly we performed 2 μs of simulation to assess the stability of the system. The electrostatic/coulombic interactions were shifted to zero between 0 and 12 Å and the Lennard-Jones interactions between 9 and 12 Å. A Berendsen thermostat with a reference temperature of 310 K in combination with a Berendsen barostat with a coupling constant of 1.0 ps, a compressibility of 5.0×10^{-6} bar^{-1}, and a reference pressure of 1 bar was used in the equivalent protocols published recently (Kalli et al., 2011, 2013). The integration step was 40 fs. For the CG simulations of monomers and dimers we used the same parameters as for the single FN2 domain simulations with a coupling constant of 1.0 ps, a compressibility of 3.0×10^{-4} bar^{-1}, and a reference pressure of 1 bar to be consistent with recent CG simulations of large membranes (Ingólfsson et al., 2014; van Eerden et al., 2015). The integration step was 20 fs.

AT MD Simulations

Atomistic simulations were carried out using the GROMOS96 43a1 force field (Scott et al., 1999). Water and counterion molecules were added to equilibrate the system. Then a 5,000-step steepest descent minimization was performed followed by an equilibration phase. The production run was then performed for 300 ns. Long-range electrostatics (beyond 10 Å) were modeled using the particle mesh Ewald procedure. The same cut-off distance was used to model van der Waals interactions. The reference temperature was 310 K. The simulation was performed at constant temperature, pressure, and particle number using semi-isotropic pressure coupling with the Parrinello-Rahman barostat (Parrinello and Rahman, 1981) and the V-rescale thermostat (Bussi et al., 2007). The integration time step was 2 fs.

Simulation Analysis

VMD (Humphrey et al., 1996) was used to render system structures and was combined with Tcl scripts to analyze the simulations. Each simulation was centered on the protein using the trjconv command provided by the GROMACS 4.5.5 package. We used Tcl scripts in VMD and take a frame every 2 ns to calculate the number of contacts for each residue. We then concatenated the results of each simulation and normalized them to obtain the graphs in Figures 3 and S3. We used the VMD plugin to perform radial distribution calculation using NC3 beads for PC and GLH beads for PG lipids. We used the Volmap VMD plugin on the last 200 ns of simulation to create the density plot shown in Figure 2B.

FN2 Expression and Mutagenesis

Cloning

A construct coding for human EphA2 FN2 (residues 436–534, Uniprot: P29317) was cloned into the AgeI-KpnI cloning site of a pHLsec vector (Aricescu et al., 2006), also coding for an N-terminal secretion signal sequence and a C-terminal polyhistidine tag. Point mutants (K441E + R443E) were generated using PCR techniques.

Protein Purification

We expressed EphA2 FN2 (wild-type or mutant) transiently in HEK293T cells, in the presence of kiffunensine (Aricescu et al., 2006). Proteins were purified from cell culture medium using Ni-affinity chromatography. EphA2 FN2 domain does not contain native glycosylation sites, but the sequence of the vector tag, together with the last residue in our construct (N534), introduced an artificial glycosylation site at N534. This site was glycosylated in the wild-type and mutant proteins (not shown). To remove the artificial glycan, we treated our samples with EndoH (Grueninger-Leitch et al., 1996), and re-purified the proteins using size-exclusion chromatography.

Liposome Pull-Down Assay

We prepared lipid vesicles by drying dioleoylphosphatidylcholine (DOPC), dioleoylphosphatidylglycerol (DOPG), or a 1:1 (w/w) mixture of DOPG and DOPC under constant argon flow to avoid oxidation. We re-suspended the resultant lipid film in buffer (100 mM NaCl, 20 mM Tris [pH 7.5]) and sonicated the mixture for 15 min to generate vesicles. Final lipid concentrations were 2 mg/ml. We mixed 100 μl of vesicle suspension with equal amounts of wild-type or mutant EphA2 FN domain 2 protein, incubated these at room temperature for 10 min, and centrifuged them at >20,000 × *g* for 10 min. We repeated the experiments seven times. For each set the bound protein fractions were visualized by western blot using mouse anti-pentaHis antibody (Qiagen). Data analysis was done with ImageJ (Schneider et al., 2012). In each set, we normalized the data using the corresponding value measured for wild-type protein pelleted with DOPC vesicles. After normalization, we calculated averages and SEM from all experiments.

SUPPLEMENTAL INFORMATION

Supplemental Information includes Supplemental Information, 3D Molecular Model, six figures, and one table and can be found with this article online at http://dx.doi.org/10.1016/j.str.2015.11.008.

AUTHOR CONTRIBUTIONS

M.C. performed and analyzed simulations. E.S. performed experiments. M.C., E.S., E.Y.J., and M.S.P.S. designed the research and together wrote the manuscript.

ACKNOWLEDGMENTS

Our thanks to Dr Antreas Kalli for advice. M.S.P.S. was supported by the Wellcome Trust (WT092970MA). This work was in part performed using the ARCHER supercomputer via the EPSRC-funded HECBioSim consortium. E.Y.J. was supported by Cancer Research UK (C375/A10976**)** and E.S. by the UK Medical Research Council (MR/L018039/1). The Wellcome Trust Center for Human Genetics is supported by the Wellcome Trust (090532/Z/09/Z).

Received: June 25, 2015
Revised: October 19, 2015
Accepted: November 13, 2015
Published: December 24, 2015

REFERENCES

Anderson, R.G., and Jacobson, K. (2002). A role for lipid shells in targeting proteins to caveolae, rafts, and other lipid domains. Science *296*, 1821–1825.

Aricescu, A.R., Lu, W., and Jones, E.Y. (2006). A time- and cost-efficient system for high-level protein production in mammalian cells. Acta Crystallogr. D Biol. Crystallogr. *62*, 1243–1250.

Arish, M., Husein, A., Kashif, M., Sandhu, P., Hasnain, S.E., Akhter, Y., and Rub, A. (2015). Orchestration of membrane receptor signaling by membrane lipids. Biochimie *113*, 111–124.

Arkhipov, A., Shan, Y., Das, R., Endres, N.F., Eastwood, M.P., Wemmer, D.E., Kuriyan, J., and Shaw, D.E. (2013). Architecture and membrane interactions of the EGF receptor. Cell *152*, 557–569.

Arkhipov, A., Shan, Y., Kim, E.T., and Shaw, D.E. (2014). Membrane interaction of bound ligands contributes to the negative binding cooperativity of the EGF receptor. PLoS Comput. Biol. *10*, e1003742.

Baker, N.A. (2001). Electrostatics of nanosystems: application to microtubules and the ribosome. Proc. Natl. Acad. Sci. USA *98*, 10037–10041.

Biggin, P.C., and Bond, P.J. (2015). Molecular dynamics simulations of membrane proteins. Methods Mol. Biol. *1215*, 91–108.

Bocharov, E.V., Mayzel, M.L., Volynsky, P.E., Goncharuk, M.V., Ermolyuk, Y.S., Schulga, A.A., Artemenko, E.O., Efremov, R.G., and Arseniev, A.S. (2008). Spatial structure and pH-dependent conformational diversity of dimeric transmembrane domain of the receptor tyrosine kinase EphA1. J. Biol. Chem. *283*, 29385–29395.

Bocharov, E.V., Mayzel, M.L., Volynsky, P.E., Mineev, K.S., Tkach, E.N., Ermolyuk, Y.S., Schulga, A.A., Efremov, R.G., and Arseniev, A.S. (2010). Left-handed dimer of EphA2 transmembrane domain: helix packing diversity among receptor tyrosine kinases. Biophys. J. *98*, 881–889.

Bond, P.J., and Sansom, M.S.P. (2006). Insertion and assembly of membrane proteins via simulation. J. Am. Chem. Soc. *128*, 2697–2704.

Bond, P.J., Holyoake, J., Ivetac, A., Khalid, S., and Sansom, M.S.P. (2007). Coarse-grained molecular dynamics simulations of membrane proteins and peptides. J. Struct. Biol. *157*, 593–605.

Bond, P.J., Wee, C.L., and Sansom, M.S.P. (2008). Coarse-grained molecular dynamics simulations of the energetics of helix insertion into a lipid bilayer. Biochemistry *47*, 11321–11331.

Bowden, T.A., Aricescu, A.R., Nettleship, J.E., Siebold, C., Rahman-Huq, N., Owens, R.J., Stuart, D.I., and Jones, E.Y. (2009). Structural plasticity of eph receptor A4 facilitates cross-class ephrin signaling. Structure *17*, 1386–1397.

Bussi, G., Donadio, D., and Parrinello, M. (2007). Canonical sampling through velocity rescaling. J. Chem. Phys. *126*, 014101.

Carvalho, R.F., Beutler, M., Marler, K.J.M., Knöll, B., Becker-Barroso, E., Heintzmann, R., Ng, T., and Drescher, U. (2006). Silencing of EphA3 through a cis interaction with ephrinA5. Nat. Neurosci. *9*, 322–330.

Chakraborty, S., Veettil, M.V., Bottero, V., and Chandran, B. (2012). Kaposi's sarcoma-associated herpesvirus interacts with EphrinA2 receptor to amplify signaling essential for productive infection. Proc. Natl. Acad. Sci. USA *109*, E1163–E1172.

Chavent, M., Chetwynd, A.P., Stansfeld, P.J., and Sansom, M.S.P. (2014). Dimerization of the EphA1 receptor tyrosine kinase transmembrane domain: insights into the mechanism of receptor activation. Biochemistry *53*, 6641–6652.

Coskun, Ü., Grzybek, M., Drechsel, D., and Simons, K. (2011). Regulation of human EGF receptor by lipids. Proc. Natl. Acad. Sci. USA *108*, 9044–9048.

Davis, T.L., Walker, J.R., Loppnau, P., Butler-Cole, C., Allali-Hassani, A., and Dhe-Paganon, S. (2008). Autoregulation by the juxtamembrane region of the human ephrin receptor tyrosine kinase A3 (EphA3). Structure *16*, 873–884.

Eswar, N., Webb, B., Marti-Renom, M.A., Madhusudhan, M.S., Eramian, D., Shen, M.-Y., Pieper, U., and Sali, A. (2007). Comparative protein structure modeling using MODELLER. Curr. Protoc. Protein Sci., Chapter 2, Unit 2.9.

Falivelli, G., Lisabeth, E.M., Rubio de la Torre, E., Perez-Tenorio, G., Tosato, G., Salvucci, O., and Pasquale, E.B. (2013). Attenuation of Eph receptor kinase activation in cancer cells by coexpressed ephrin ligands. PLoS One *8*, e81445.

Franco-Gonzalez, J.F., Ramos, J., Cruz, V.L., and Martinez-Salazar, J. (2014). Exploring the dynamics and interaction of a full ErbB2 receptor and Trastuzumab-Fab antibody in a lipid bilayer model using Martini coarse-grained force field. J. Comput. Aided Mol. Des. *28*, 1093–1107.

Gauthier, L.R., and Robbins, S.M. (2003). Ephrin signaling: one raft to rule them all? One raft to sort them? One raft to spread their call and in signaling bind them? Life Sci. *74*, 207–216.

Grueninger-Leitch, F., D'Arcy, A., D'Arcy, B., and Chène, C. (1996). Deglycosylation of proteins for crystallization using recombinant fusion protein glycosidases. Protein Sci. *5*, 2617–2622.

Herbert, S.P., and Stainier, D.Y. (2011). Molecular control of endothelial cell behaviour during blood vessel morphogenesis. Nat. Rev. Mol. Cell Biol. *12*, 551–564.

Hess, B., Kutzner, C., Van Der Spoel, D., and Lindahl, E. (2008). GROMACS 4: algorithms for highly efficient, load-balanced, and scalable molecular simulation. J. Chem. Theory Comput. *4*, 435–447.

Himanen, J.P. (2011). Ectodomain structures of Eph receptors. Semin. Cell Dev. Biol *23*, 35–42.

Himanen, J.-P., Chumley, M.J., Lackmann, M., Li, C., Barton, W.A., Jeffrey, P.D., Vearing, C., Geleick, D., Feldheim, D.A., Boyd, A.W., et al. (2004). Repelling class discrimination: ephrin-A5 binds to and activates EphB2 receptor signaling. Nat. Neurosci. *7*, 501–509.

Himanen, J.P., Yermekbayeva, L., Janes, P.W., Walker, J.R., Xu, K., Atapattu, L., Rajashankar, K.R., Mensinga, A., Lackmann, M., Nikolov, D.B., et al. (2010). Architecture of Eph receptor clusters. Proc. Natl. Acad. Sci. USA *107*, 10860–10865.

Hofman, E.G., Ruonala, M.O., Bader, A.N., van den Heuvel, D., Voortman, J., Roovers, R.C., Verkleij, A.J., Gerritsen, H.C., and van Bergen en Henegouwen, P.M.P. (2008). EGF induces coalescence of different lipid rafts. J. Cell. Sci. *121*, 2519–2528.

Humphrey, W., Dalke, A., and Schulten, K. (1996). VMD: visual molecular dynamics. J. Mol. Graph *14*, 33–38, 27–28.

Ingólfsson, H.I., Melo, M.N., van Eerden, F.J., Arnarez, C., Lopez, C.A., Wassenaar, T.A., Periole, X., de Vries, A.H., Tieleman, D.P., and Marrink, S.J. (2014). Lipid organization of the plasma membrane. J. Am. Chem. Soc. *136*, 14554–14559.

Jaud, S., Tobias, D.J., Falke, J.J., and White, S.H. (2007). Self-induced docking site of a deeply embedded peripheral membrane protein. Biophys. J. *92*, 517–524.

Jefferys, E., Sands, Z.A., Shi, J., Sansom, M.S.P., and fowler, P.W. (2015). Alchembed: a computational method for incorporating multiple proteins into complex lipid geometries. J. Chem. Theory Comput. *11*, 2743–2754.

Kalli, A.C., and Sansom, M.S.P. (2014). Interactions of peripheral proteins with model membranes as viewed by molecular dynamics simulations. Biochem. Soc. Trans. *42*, 1418–1424.

Kalli, A.C., Wegener, K.L., Goult, B.T., Anthis, N.J., Campbell, I.D., and Sansom, M.S.P. (2010). The structure of the talin/integrin complex at a lipid bilayer: an NMR and MD simulation study. Structure *18*, 1280–1288.

Kalli, A.C., Campbell, I.D., and Sansom, M.S.P. (2011). Multiscale simulations suggest a mechanism for integrin inside-out activation. Proc. Natl. Acad. Sci. USA *108*, 11890–11895.

Kalli, A.C., Morgan, G., and Sansom, M.S.P. (2013). Interactions of the auxilin-1 PTEN-like domain with model membranes result in nanoclustering of phosphatidyl inositol phosphates. Biophys. J. *105*, 137–145.

Kalli, A.C., Devaney, I., and Sansom, M.S.P. (2014). Interactions of phosphatase and tensin homologue (PTEN) proteins with phosphatidylinositol phosphates: insights from molecular dynamics simulations of PTEN and voltage sensitive phosphatase. Biochemistry *53*, 1724–1732.

Kästner, J., Loeffler, H.H., Roberts, S.K., Martin-Fernandez, M.L., and Winn, M.D. (2009). Ectodomain orientation, conformational plasticity and oligomerization of ErbB1 receptors investigated by molecular dynamics. J. Struct. Biol. *167*, 117–128.

Kaszuba, K., Grzybek, M., Orłowski, A., Danne, R., Róg, T., Simons, K., Coskun, Ü., and Vattulainen, I. (2015). N-Glycosylation as determinant of epidermal growth factor receptor conformation in membranes. Proc. Natl. Acad. Sci. USA *112*, 4334–4339.

Koldsø, H., Shorthouse, D., Hélie, J., and Sansom, M.S.P. (2014). Lipid clustering correlates with membrane curvature as revealed by molecular simulations of complex lipid bilayers. PLoS Comput. Biol. *10*, e1003911.

Kubiak-Ossowska, K., Mulheran, P.A., and Nowak, W. (2014). Fibronectin module FN III9 adsorption at contrasting solid model surfaces studied by atomistic molecular dynamics. J. Phys. Chem. B *118*, 9900–9908.

Kullander, K., and Klein, R. (2002). Mechanisms and functions of eph and ephrin signalling. Nat. Rev. Mol. Cell Biol. *3*, 475–486.

Lai, K.-O., and Ip, N.Y. (2009). Synapse development and plasticity: roles of ephrin/Eph receptor signaling. Curr. Opin. Neurobiol. *19*, 275–283.

Lee, H.J., Hota, P.K., Chugha, P., Guo, H., Miao, H., Zhang, L., Kim, S.-J., Stetzik, L., Wang, B.-C., and Buck, M. (2012). NMR structure of a heterodimeric SAM: SAM complex: characterization and manipulation of EphA2 binding reveal new cellular functions of SHIP2. Structure *20*, 41–55.

Levental, I., Grzybek, M., and Simons, K. (2010). Greasing their way: lipid modifications determine protein association with membrane rafts. Biochemistry *49*, 6305–6316.

Li, J., Wen, P.-C., Moradi, M., and Tajkhorshid, E. (2015). Computational characterization of structural dynamics underlying function in active membrane transporters. Curr. Opin. Struct. Biol. *31*, 96–105.

Lohmüller, T., Xu, Q., and Groves, J.T. (2013). Nanoscale obstacle arrays frustrate transport of EphA2-Ephrin-A1 clusters in cancer cell lines. Nano Lett. *13*, 3059–3064.

Lumb, C.N., and Sansom, M.S.P. (2012). Finding a needle in a haystack: the role of electrostatics in target lipid recognition by PH domains. PLoS Comput. Biol. *8*, e1002617.

Marrink, S.J., and Tieleman, D.P. (2013). Perspective on the Martini model. Chem. Soc. Rev. *42*, 6801–6822.

Monticelli, L., Kandasamy, S.K., Periole, X., Larson, R.G., Tieleman, D.P., and Marrink, S.-J. (2008). The MARTINI coarse-grained force field: extension to proteins. J. Chem. Theory Comput. *4*, 819–834.

Nikolov, D.B., Xu, K., and Himanen, J.P. (2014). Homotypic receptor-receptor interactions regulating Eph signaling. Cell Adh. Migr. *8*, 360–365.

Parrinello, M., and Rahman, A. (1981). Polymorphic transitions in single crystals: a new molecular dynamics method. J. Appl. Phys. *52*, 7182.

Pasquale, E.B. (2010). Eph receptors and ephrins in cancer: bidirectional signalling and beyond. Nat. Rev. Cancer *10*, 165–180.

Periole, X., Cavalli, M., Marrink, S.-J., and Ceruso, M.A. (2009). Combining an elastic network with a coarse-grained molecular force field: structure, dynamics, and intermolecular recognition. J. Chem. Theory Comput. *5*, 2531–2543.

Pike, L.J., Han, X., Chung, K.-N., and Gross, R.W. (2002). Lipid rafts are enriched in arachidonic acid and plasmenylethanolamine and their composition is independent of caveolin-1 expression: a quantitative electrospray ionization/mass spectrometric analysis. Biochemistry *41*, 2075–2088.

Posse de Chaves, E., and Sipione, S. (2010). Sphingolipids and gangliosides of the nervous system in membrane function and dysfunction. FEBS Lett. *584*, 1748–1759.

Qin, H., Noberini, R., Huan, X., Shi, J., Pasquale, E.B., and Song, J. (2010). Structural characterization of the EphA4-Ephrin-B2 complex reveals new features enabling Eph-ephrin binding promiscuity. J. Biol. Chem. *285*, 644–654.

Roberts, S.K., Tynan, C.J., Winn, M., and Martin-Fernandez, M.L. (2012). Investigating extracellular in situ EGFR structure and conformational changes using FRET microscopy. Biochem. Soc. Trans. *40*, 189–194.

Schneider, C.A., Rasband, W.S., and Eliceiri, K.W. (2012). NIH image to ImageJ: 25 years of image analysis. Nat. Methods *9*, 671–675.

Scott, W.R.P., Hünenberger, P.H., Tironi, I.G., Mark, A.E., Billeter, S.R., Fennen, J., Torda, A.E., Huber, T., Krüger, P., and Van Gunsteren, W.F. (1999). The GROMOS biomolecular simulation program package. J. Phys. Chem. A *103*, 3596–3607.

Seiradake, E., Harlos, K., Sutton, G., Aricescu, A.R., and Jones, E.Y. (2010). An extracellular steric seeding mechanism for Eph-ephrin signaling platform assembly. Nat. Struct. Mol. Biol. *17*, 398–402.

Seiradake, E., Schaupp, A., del Toro Ruiz, D., Kaufmann, R., Mitakidis, N., Harlos, K., Aricescu, A.R., Klein, R., and Jones, E.Y. (2013). Structurally encoded intraclass differences in EphA clusters drive distinct cell responses. Nat. Struct. Mol. Biol. *20*, 958–964.

Simons, K., and Toomre, D. (2000). Lipid rafts and signal transduction. Nat. Rev. Mol. Cell Biol. *1*, 31–39.

Singh, D.R., Ahmed, F., King, C., Gupta, N., Salotto, M., Pasquale, E.B., and Hristova, K. (2015). EphA2 unliganded dimers suppress EphA2 pro-tumorigenic signaling. J. Biol. Chem. *290*, 27271–27279.

Stansfeld, P.J., and Sansom, M.S.P. (2011a). From coarse grained to atomistic: a serial multiscale approach to membrane protein simulations. J. Chem. Theory Comput. *7*, 1157–1166.

Stansfeld, P.J., and Sansom, M.S.P. (2011b). Molecular simulation approaches to membrane proteins. Structure *19*, 1562–1572.

Stapleton, D., Balan, I., Pawson, T., and Sicheri, F. (1999). The crystal structure of an Eph receptor SAM domain reveals a mechanism for modular dimerization. Nat. Struct. Biol. *6*, 44–49.

Sun, F., Xu, L., Chen, P., Wei, P., Qu, J., Chen, J., and Luo, S.-Z. (2015). Insights into the packing switching of the EphA2 transmembrane domain by molecular dynamic simulations. J. Phys. Chem. B *119*, 7816–7824.

Tawadros, T., Brown, M.D., Hart, C.A., and Clarke, N.W. (2012). Ligand-independent activation of EphA2 by arachidonic acid induces metastasis-like behaviour in prostate cancer cells. Br. J. Cancer *107*, 1737–1744.

van Eerden, F.J., de Jong, D.H., de Vries, A.H., Wassenaar, T.A., and Marrink, S.J. (2015). Characterization of thylakoid lipid membranes from cyanobacteria and higher plants by molecular dynamics simulations. Biochim. Biophys. Acta *1848*, 1319–1330.

van Meer, G., Voelker, D.R., and Feigenson, G.W. (2008). Membrane lipids: where they are and how they behave. Nat. Rev. Mol. Cell Biol. *9*, 112–124.

Webb, S.E.D., Roberts, S.K., Needham, S.R., Tynan, C.J., Rolfe, D.J., Winn, M.D., Clarke, D.T., Barraclough, R., and Martin-Fernandez, M.L. (2008). Single-molecule imaging and fluorescence lifetime imaging microscopy show different structures for high- and low-affinity epidermal growth factor receptors in A431 cells. Biophys. J. *94*, 803–819.

Wiesner, S., Wybenga-Groot, L.E., Warner, N., Lin, H., Pawson, T., Forman-Kay, J.D., and Sicheri, F. (2006). A change in conformational dynamics underlies the activation of Eph receptor tyrosine kinases. EMBO J. *25*, 4686–4696.

Wybenga-Groot, L.E., Baskin, B., Ong, S.H., Tong, J., Pawson, T., and Sicheri, F. (2001). Structural basis for autoinhibition of the Ephb2 receptor tyrosine kinase by the unphosphorylated juxtamembrane region. Cell *106*, 745–757.

Xu, K., Tzvetkova-Robev, D., Xu, Y., Goldgur, Y., Chan, Y.-P., Himanen, J.P., and Nikolov, D.B. (2013). Insights into Eph receptor tyrosine kinase activation from crystal structures of the EphA4 ectodomain and its complex with ephrin-A5. Proc. Natl. Acad. Sci. USA *110*, 14634–14639.

Structure, Volume *24*

Supplemental Information

Structures of the EphA2 Receptor at the Membrane: Role of Lipid Interactions

Matthieu Chavent, Elena Seiradake, E. Yvonne Jones, and Mark S.P. Sansom

Supplemental Information

Supplementary text related to Method and Figures 2, 6, S3, S4 and S6

In order to quantify the protein-lipids interactions occurring during the simulations, we first define an interaction count function, $C_l(res_i)$of the interactions between residue i and the lipid type l for a time *t*:

$$C_l(res_i) = \begin{cases} \text{Nb. atoms from lipid type } l \text{ within 8 Å of } res_i \\ 0 \text{ otherwise} \end{cases}$$

The proportional interaction of a residue i for a lipid type l , $I_{i,l}$, is then obtained summation of the interaction counts over the time the protein forms a stable interaction with the membrane and normalising it by the duration of the interaction:

$$I_{i,l} = \frac{1}{N - n_b} \sum_{n_b}^{N} C_l(res_i)$$

where N is the total simulation time and n_b the time at which the interaction occurs.

This allows one to define a proportional interaction for PC and PG lipids (respectively $I_{i,POPC}$ and $I_{i,POPG}$) for a residue i.

We then normalized over the different residues using the maximum of PC+PG value to obtain a value between 0 and 1:

$$Max_{I(PC,PG)} = \max(I_{i,POPC} + I_{i,POPG})$$

with i varying from first residue number to the last.

$$N(I_i) = \frac{I_{i,POPC}}{Max_{I(PC,PG)}} + \frac{I_{i,POPG}}{Max_{I(PC,PG)}}$$

We used $N(I_i)$ values to create the histograms presented in Figures 2, 6, S3, S4 and S6.

The curve, highlighting the preference for PG lipid was obtained using the expression:

$$P_{i,POPG} = \frac{I_{i,POPG}}{Max_{I(PC,PG)}} - \frac{I_{i,POPC}}{Max_{I(PC,PG)}}$$

Table S1 related to Table 1: Summary of the FN2 simulations interacting with the membrane. Simulation duration and time to binding for the diffusion protocol (Wild Type – WT – and mutant). The 'time to binding' refers to the time elapsed between the start of the simulation and the onset of a stable interaction with the membrane. Each simulation was run for at least 500 ns of simulation following formation of this initial interaction. If the protein did not interact with the the membrane after 6 μs it was considered not to bind.

System name	duration (ns)	binding	time to binding (ns)
**100% PC (WT)**			
FN2 180°	6000	No	###
FN2 0°	6000	No	###
FN2 90° A	6000	No	###
FN2 90° B	6000	yes	1430
**60% PC - 40% PG (FN2 WT)**			
FN2 180°	2000	yes	1525
FN2 0°	2000	yes	1500
FN2 90° A	1000	yes	147
FN2 90° B	1000	yes	107
**60% PC - 40% PG (FN2 K441E+K443E mutant)**			
FN2 180°	1000	Yes	324
FN2 0°	3000	Yes	1934
FN2 90° A	2000	Yes	1400
FN2 90° B	3000	Yes	2150
**60% PC - 40% PG (FN2 K441E+K443E+R465E mutant)**			
FN2 180°	6000	No	###
FN2 0°	1000	Yes	343
FN2 90° A	5000	Yes	4198
FN2 90° B	2000	Yes	990
**60% PC - 40% PG (FN2 K441E+K443E+R465E+F490A mutant)**			
FN2 180°	2000	Yes	1672
FN2 0°	4000	Yes	3121
FN2 90° A	6000	No	###
FN2 90° B	2000	No	###
**60% PC - 40% PG (FN2 K441E+K443E+R465E+F490A+W467A mutant)**			
FN2 180°	2000	Yes	1498
FN2 0°	7000	Yes	5759
FN2 90° A	7000	Yes	5995
FN2 90° B	2000	Yes	1095

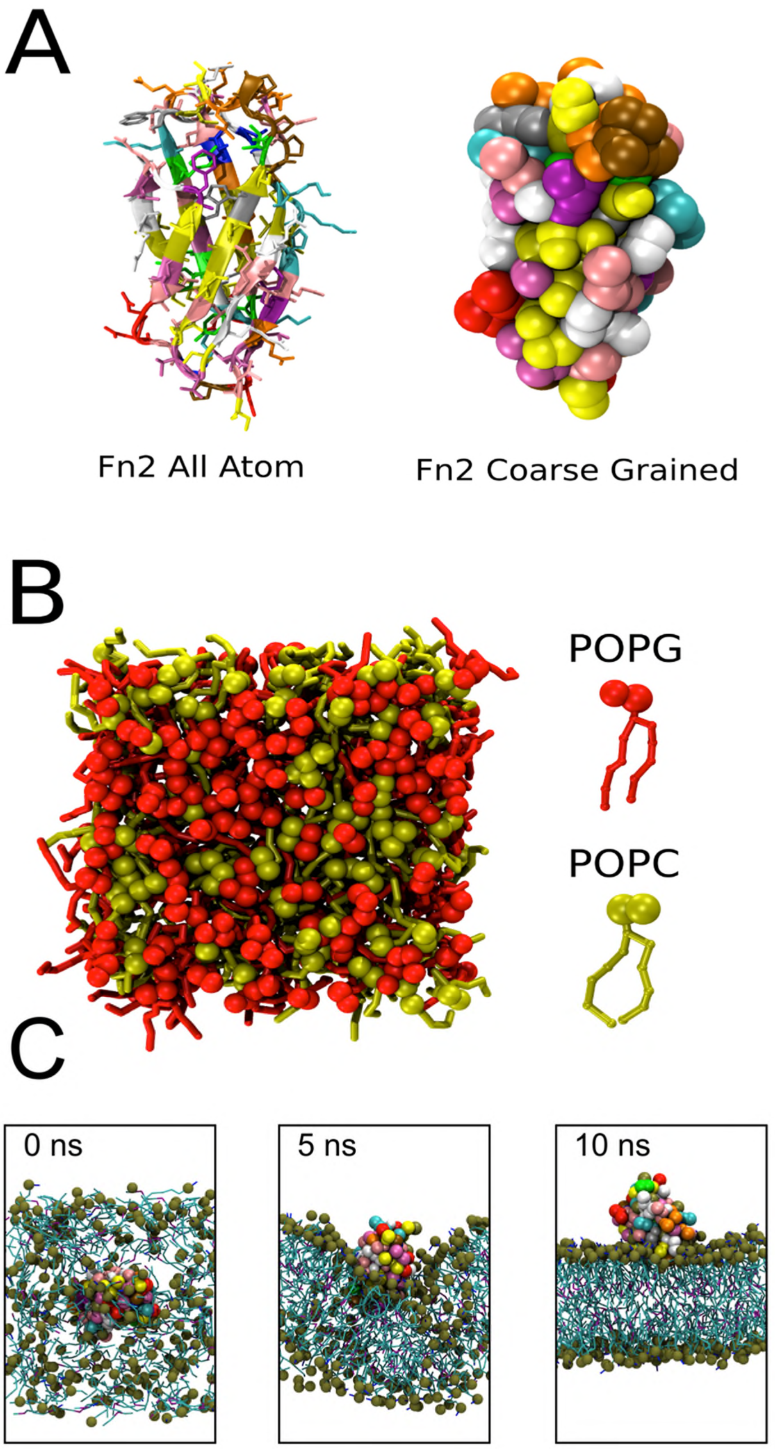

Figure S1 (related to Figure 1 and Methods): (A) Atomistic (left) and coarse-grained (right) models of the FN2 domain. (B) Mixed (PG+PC) membrane model for CG simulations. (C) Progress of the bilayer self-assembly simulation at different time steps.

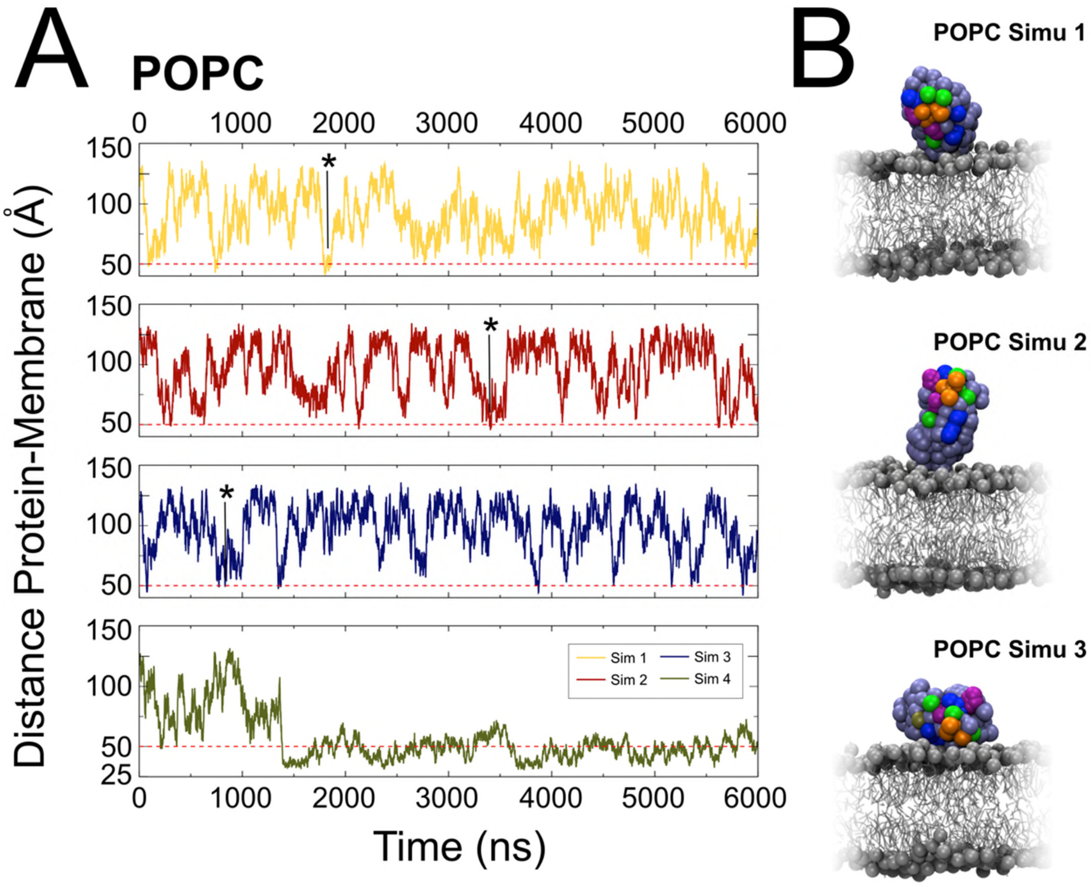

Figure S2 (related to Figure 1): distance between the FN2 domain and the membrane during CG simulations. (A) Evolution of the distance between the centres of mass of the membrane and of the FN2 domain in function of the time for CG simulations performed with the PC bilayer. (B) Different transient interactions of the FN2 domain with the PC membrane (highlighted by a star in A). Residues coloured in green, violet dark blue and orange are the main interacting residues depicted Fig. 2 A and S2. In (A) the dashed line depicts the distance from which the protein can touch the membrane, and in (B) Simu 1, Simu 2 and Simu 3 correspond to snapshots from the respective repeat simulations

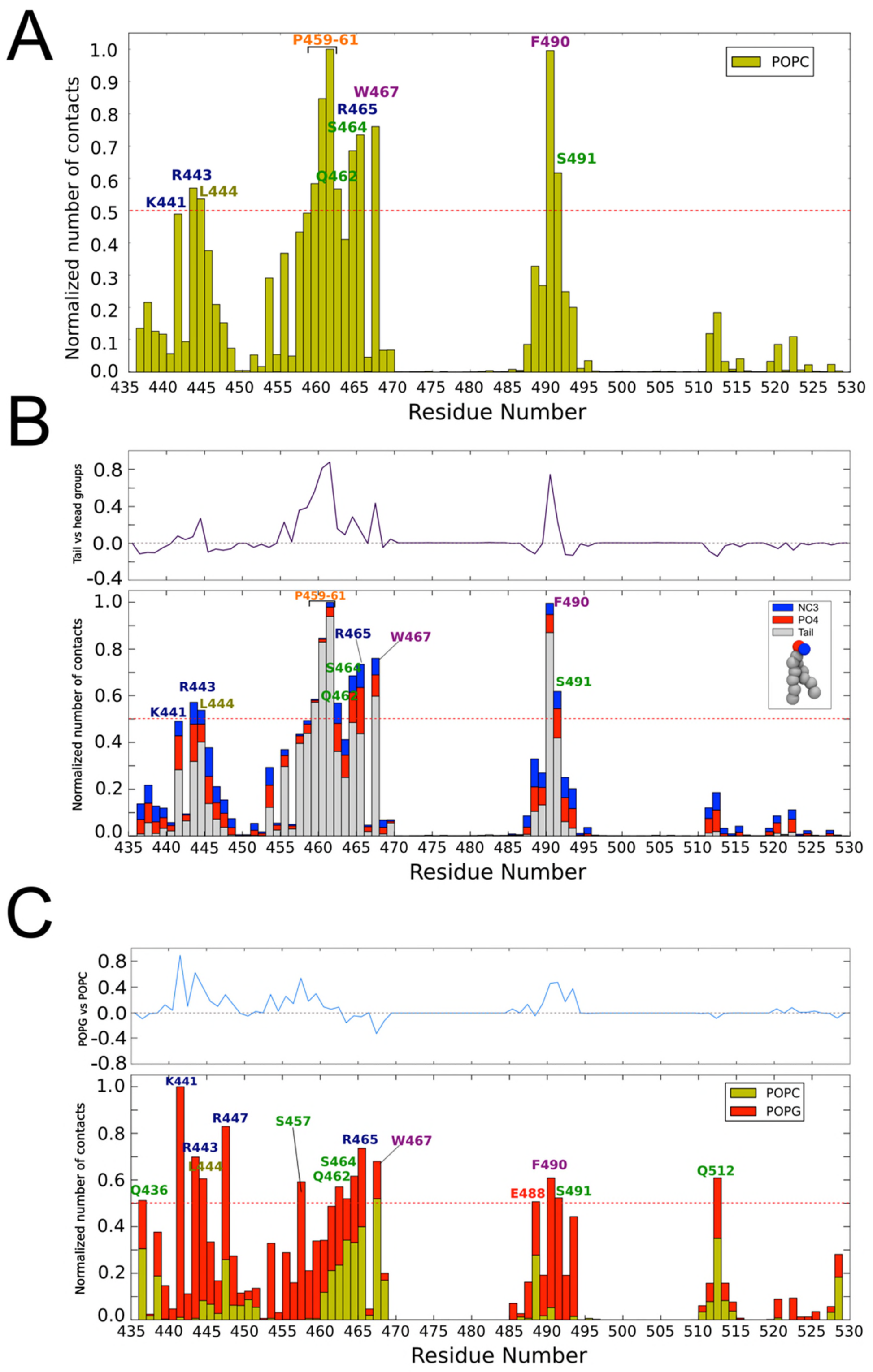

Figure S3 (related to Figure 1): Normalized averaged number of contacts. (A) Normalized average number of contacts of PC lipids as a function of residue number for the Wild Type. (B) Decomposition of the normalized average number of contacts for PC between NC3, PO4 particles and lipid tail. Dark curve displays the difference between the number of contact for the head group particles (NC3 and PO4) and the tail: positive values depict the preference of the residue for the tail. (C) Normalized average number of contacts of PC-PG membrane with the FN2 WT as a function of residue number for the AT simulations. The blue curve displays the difference between the number of contact for the PG lipids and PC lipids: positive values depict the preference of the residue for the PG lipids.

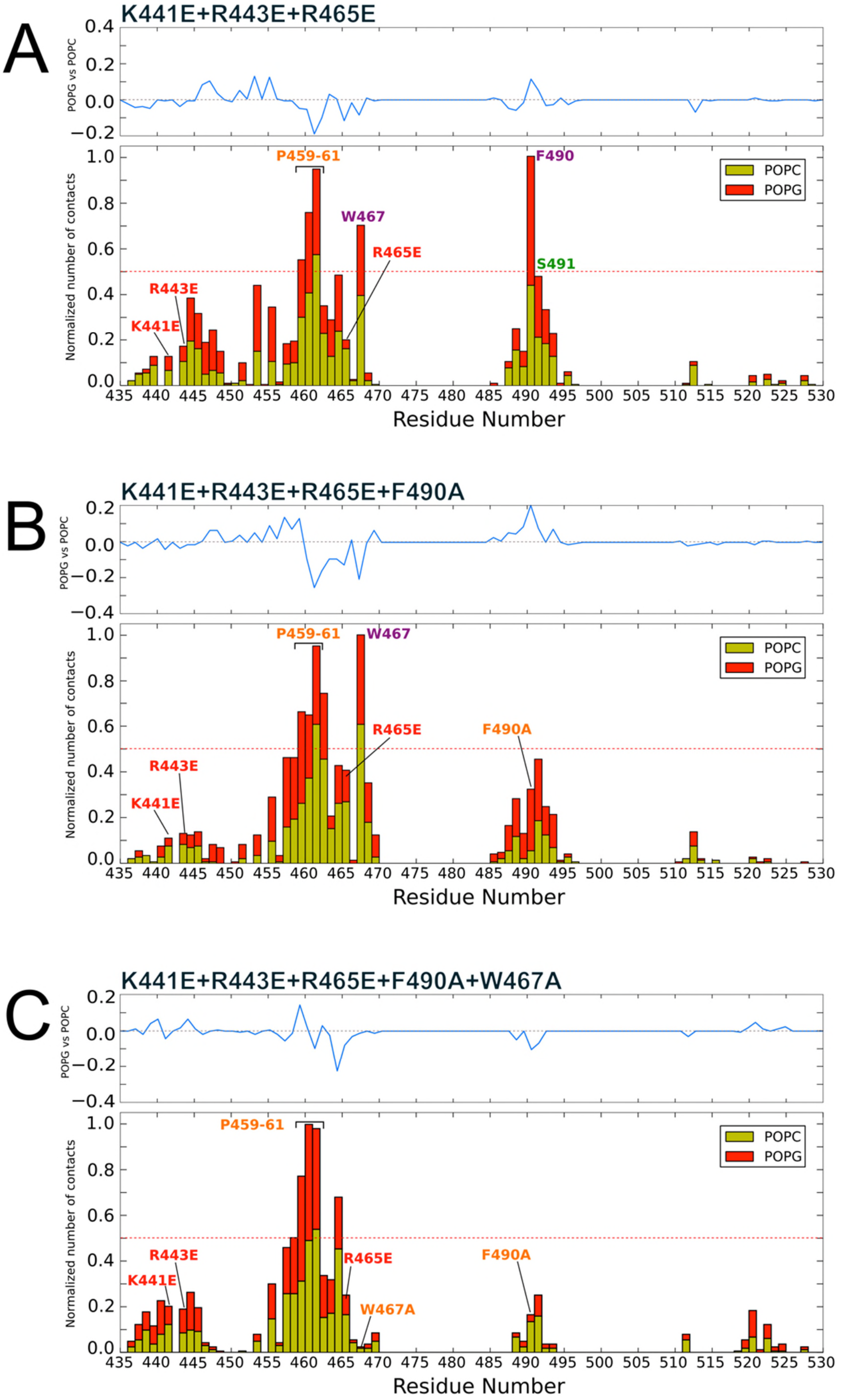

Figure S4 (related to Figure 2): Normalized averaged number of contacts for different mutants. Normalized average number of contacts of a PC-PG membrane with the (A) FN2 K441+R443+R365, (B) K441+R443+R365+F490A, and (C) K441+R443+R365 +F490A+W467A mutants as a function of residue number for the CG simulations. The blue curve displays the difference between the number of contact for the PG lipids and PC lipids: positive values depict the preference of the residue for the PG lipids.

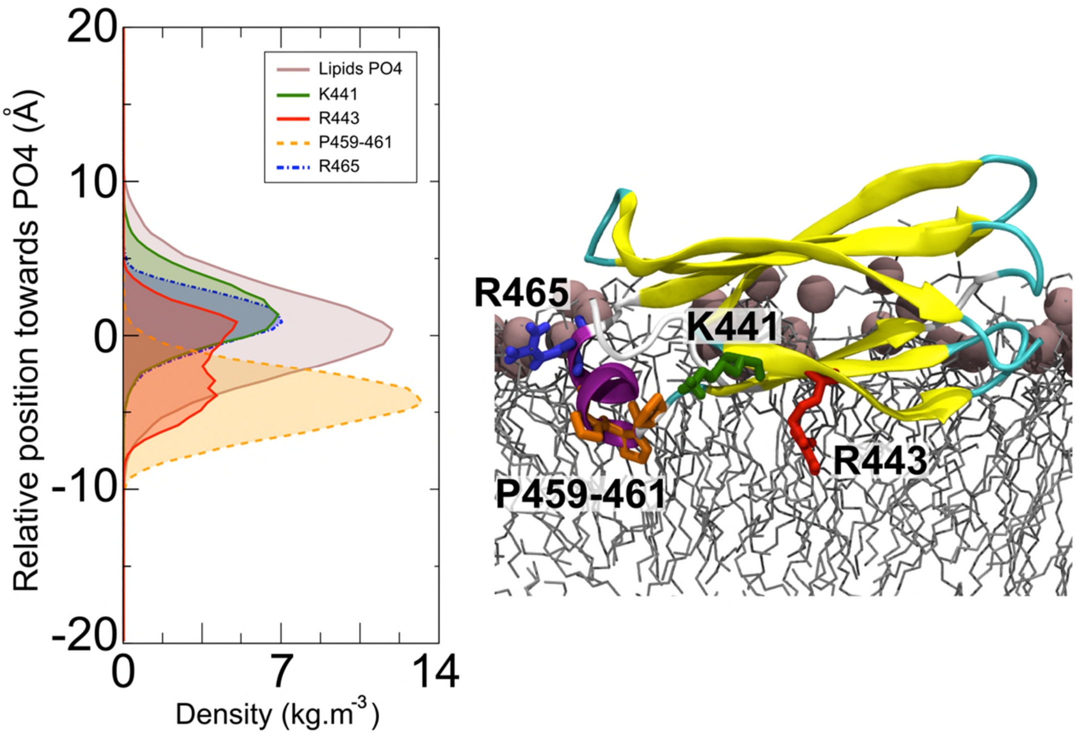

Figure S5 (related to Figure 4): FN2 positioning at the membrane. On left, the graph depicts the density profiles along the bilayer normal of several important residues for the interaction with the membrane. The profile is centred on the PO4 headgroup density. The amplitude of PO4 curve was divided by ten to fit in the graph. On right, a snapshot taken at the end of the AT simulation illustrates the graph results.

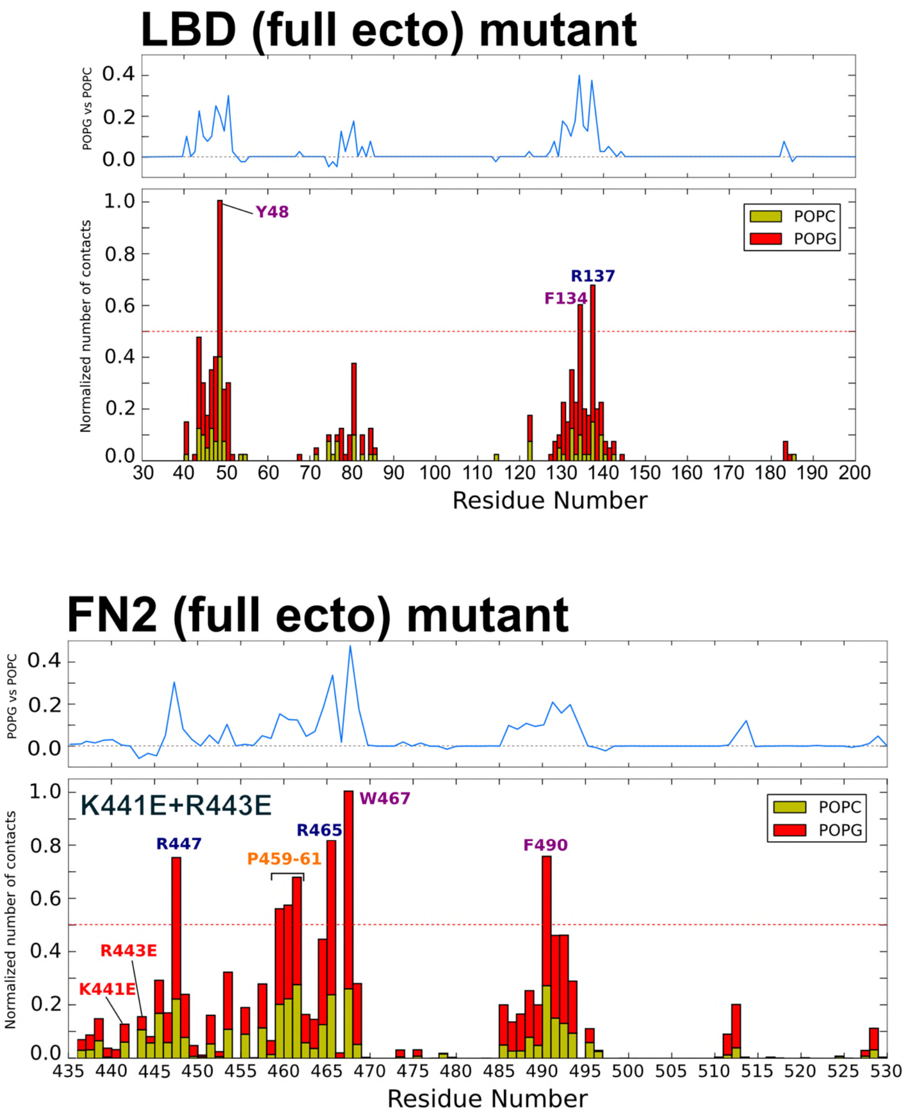

Figure S6 (related to Figure 6): Normalized averaged number of contacts of the LBD (upper panel) and FN2 domains (lower panel) with a PC-PG membrane for the K441E+R443E full ectododmain mutant. The blue curve displays the difference between the number of contact for the PG lipids and PC lipids: positive values depict the preference of the residue for the PG lipids.

3D Molecular Model (related to Figure 4):

A model of the FN2 domain at the membrane surface linked to the TM domain. Note that to obtain the numbering presented in the paper one has to add 435 to the residue number. This model is presented in Figure 4A of the paper.
